# Emergence of relaxor-like ferroelectric nature in nanograined Pb(Zr_0.95_Ti_0.05_)O_3_ ceramic thick films for energy storage applications

**DOI:** 10.1186/s40580-025-00511-3

**Published:** 2025-09-29

**Authors:** Nirmal Prashanth Maria Joseph Raj, Hyunseok Song, Satyabrata Lenka, Geon-Tae Hwang, Dae-Yong Jeong, Mahesh Peddigari, Jungho Ryu

**Affiliations:** 1 Department of Physics, Indian Institute of Technology Hyderabad, Kandi, 502284 Telangana India; 2 School of Materials Science and Engineering, Yeungnam University, Daehak-ro, Gyeongsan-si, Gyeongsangbuk-do Republic of Korea; 3 Department of Materials Science and Engineering, Pukyong National University, 45, Yongso-ro, Nam-Gu, Busan, Republic of Korea; 4 Program in Semiconductor Convergence, Department of Materials Science and Engineering, Inha University, Incheon, Republic of Korea

## Abstract

In this study, we demonstrated the nanostructuring of the ferroelectric (FE) phase of Pb(Zr_0.95_Ti_0.05_)O_3_ (PZT-95/5) into a thick film with relaxor**-**like FE (RFE) characteristics. This transformation results in exceptionally high dielectric breakdown strength (*E*_*DBS*_) and energy storage density properties. The high kinetic energy from aerosol deposition transformed the bulk PZT-95/5 from a normal FE system into a RFE system by forming a nanostructured grain with nanodomains within a nonpolar matrix. This nanostructure enables easy domain switching, resulting in low remanent polarization. The resulting high density of grain boundaries due to nanograin formation and the nonpolar structure act as barriers to charge flow, resulting in high breakdown strength. Collectively, these effects resulted in a significantly enhanced *E*_*DBS*_ of 5.6 MV/cm and a maximum polarization of 80 µC/cm^2^. These properties, evidenced by slim hysteresis loops, demonstrate that the prepared PZT-95/5 thick film is a superior capacitive material with a high recoverable energy density of 116 J/cm^3^. Furthermore, the film exhibited reliable fatigue endurance up to 10^7^ cycles and thermal stability from room temperature to 140^°^C. The film also exhibited a peak power density of 35 MW/cm^3^ under a practical electric field of 0.45 MV/cm (180 V) and a fast discharging speed (*τ*_0.9_) of 230 ns. These properties, in addition to the minimal fabrication steps and superior capacitive characteristics, demonstrate the strong potential of the prepared PZT-95/5 thick film for use in next-generation energy storage devices.

**Supplementary Information:**

The online version contains supplementary material available at 10.1186/s40580-025-00511-3.

## Graphical Abstract

The non-relaxor in nature materials beyond compositional modification, were made into exhibit RFE like slim hysteresis loop with enhanced polarization and giant breakdown strength for the improved energy storage performance, through the mechanically tailored nanograin engineering by aerosol deposition film formation. The AD developed ~ 4 μm thick film exhibited the energy storage performance of 116 J/cm^3^.

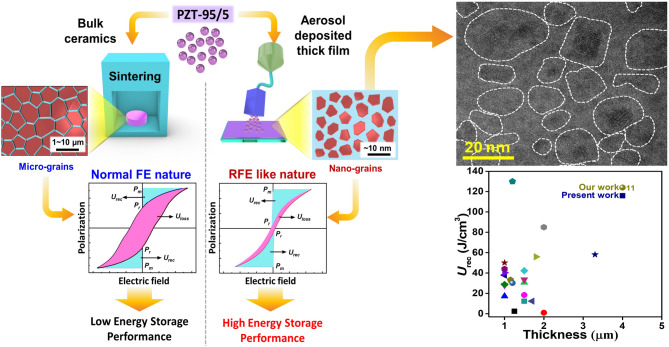

## Introduction

Recent trends in sustainable energy research demand systems that are small, adaptable, and capable of responding to multiple excitations for versatile usage and high performance. The energy storage medium is a vital component of current technologies, including renewable energy generation, smart electric vehicles, sensors, smart grids, RFID technologies, and pulsed power devices [1–3]. Although the energy storage density of electrostatic capacitors is lower than that of electrochemical supercapacitors, their high power density makes them a unanimous choice for high-power pulsed DC applications. The development of a dielectric system with superior energy density and efficiency for next-generation energy storage media is essential. The energy density of capacitors depends on the change in material polarization (*P*) under an applied electric field (*E*) and is typically determined from the ferroelectric (FE) *P-E* loops. The total energy density of a capacitor can be calculated using the equation *U*_*st*_ = $\:{\int\:}_{{P}_{0}}^{{P}_{m}}EdP$, and the recoverable energy density is given by *U*_*rec*_ = $\:{\int\:}_{{P}_{r}}^{{P}_{m}}EdP$. A slim hysteresis loop with high reversible polarization (*ΔP*), which is the difference between the maximum polarization (*P*_*m*_) and remanent polarization (*P*_*r*_), and a maximum dielectric breakdown strength (*E*_*DBS*_) is required to obtain a high energy density [4]. The efficiency (*η*) of the device can be calculated using the equation *U*_*rec*_ / *U*_*st*_.

Ferroic materials, including FEs, antiferroelectrics (AFE), and relaxor FEs (RFE), are top contenders for this usage. This is due to their multifunctional characteristics and polarization switching ability to respond to external stimuli, such as electric field, strain, and temperature. High-performance AFE and RFE systems have been studied for their multifunctional characteristics, particularly for energy storage and electrocaloric applications in film form in recent years [5–13]. Compared with bulk ferroic systems, films are preferred for certain applications due to their low voltage requirement to yield a high electric field, ease of miniaturization, and flexible device fabrication [14, 15]. Although normal FE systems (such as BaTiO_3_, Pb(Zr, Ti)O_3_ (PZT), and Bi_0.5_Na_0.5_TiO_3_) exhibit good *P*_*m*_, their *P*_*r*_ values are high, resulting in low *ΔP*, *E*_*DBS*_, and *U*_*rec*_ [16, 17]. A limited number of FE and AFE materials exhibit RFE characteristics, making them preferred for energy storage applications. Their structures, comprising nanoscale domains and polar nano regions (PNRs), require high external electric fields for domain alignment. This results in a slim hysteresis loop and a high *E*_*DBS*_, which are important parameters for energy storage applications. The RFE characteristics of a system are also distinguishable by its frequency dispersion in a dielectric phase transition peak [18]. Various techniques have been employed to obtain high-performance electrostatic energy storage capacitors, including composition engineering [19–21], multilayer effects [22, 23], microstructure engineering [24], nanograin engineering [11, 21, 25], space charge effects [26, 27], heterojunction effects [28, 29], design of high-entropy systems [30, 31], topological vortex domains [32], weakly coupled relaxor materials [33, 34], and nanoscale polarization heterogeneous structures [35–37]. For example, recently introduced heterogeneous nanoscale polarization structures provide ultrahigh polarization flexibility. This includes varying polarization orientation, submicron grain size, enhanced breakdown strength, improved polarization with relaxation behavior, and excellent energy storage performance. This approach increased the energy storage density of Bi_0.5_Na_0.5_TiO_3_ ceramic by 10 times while enhancing its energy efficiency and ultrafast discharge rate. Applied to a (1 − *x*) BaTiO_3_-*x*Bi(Ni_0.5_Zr_0.5_)O_3_ thin film capacitor, the same approach resulted in a high energy density of 103.7 J/cm^3^ and efficiency of 88.3% under an 8.3-MV/cm field [36].

Conventional FE/AFE systems have been transformed into RFE systems by the formation of PNRs, primarily via chemical heterogeneity. Although this approach improves *U*_*rec*_, it adversely affects polarization or *E*_*DBS*_. Furthermore, this approach is compositionally limited to certain materials and crystal systems [38, 39]. To overcome this challenge, a microstructure-level approach based on aerosol deposition (AD) was proposed in our previous study on Pb(Zr_0.52_Ti_0.48_)O_3_ systems. The AD process transformed normal FEs into artificial RFEs [11]. The AD technique induced RFE characteristics in the lead zirconate titanate (PZT) system to exhibit a high energy density of 124.1 J/cm^3^. The Zr-rich PZT compositions exhibit complex structural behaviors that lead to variations in electrical properties. This makes them highly useful for various applications. The Pb(Zr_0.95_Ti_0.05_)O_3_ (PZT-95/5) system with lower Zr^4+^ substitution exhibits a FE rhombohedral phase at room temperature (RT), whereas higher Zr^4+^ substitution leads to the formation of an AFE orthorhombic phase. The PZT-95/5 system is intriguing because of the coexistence of an AFE orthorhombic phase and a FE rhombohedral phase. The stability of these phases is primarily governed by the domain size, making this material a promising candidate in electrocaloric refrigeration and energy storage applications [40–42]. In the present study, the energy storage properties of PZT-95/5 after the mechanical tailoring of its domains to the film form were investigated [43]. PZT-based high-performance energy storage thick film capacitors on Pt/Ti/SiO_2_/Si substrates were fabricated using the AD technique, followed by thermal annealing at 600 °C for 1 h. The electrical properties, fatigue endurance, and thermal stability of the fabricated high-density PZT-95/5 dielectric ceramic film with a thickness of 4 μm were characterized. Thick films were selected because of their higher absolute stored energy and applicable voltage than those of thin films. In addition, these films require less voltage to develop higher electric fields than bulk systems. Overall, the selection is appropriate for storage applications.

The fabricated thick film-based capacitor exhibited a remarkable recoverable energy density of 116 J/cm^3^ and charge–discharge efficiency of over 78% due to the formation of a nanocomposite comprising mechanically tailored nanograins in the nonpolar amorphous matrix. The PZT-95/5 capacitors exhibited good, reliable fatigue endurance up to 10^7^ cycles with a slight 8% drop from the initial storage performance and temperature stability from RT to 140 °C. Furthermore, the capacitor discharged 90% of its energy density in 230 ns with an instantaneous peak power density of 35 MW/cm^3^. The high performance of these PZT-95/5 thick films, fabricated via a low-cost AD process, paves the way for next-generation energy storage devices. This process mechanically tailors a RFE nature, enabling this efficient and promising development.

## Experimental

### Synthesis of PZT-95/5 particles

The feedstock powder of Pb(Zr_0.95_Ti_0.05_)O_3_ was prepared using a solid-state reaction method. The raw materials, including lead (II) oxide (PbO), zirconium oxide (ZrO_2_), and titanium (IV) oxide (TiO_2_) with a purity of > 99.9%, were purchased from Sigma-Aldrich. These materials were used as precursors and weighed stoichiometrically. Ball milling was used to mix the weighed powders with the ethanol medium for effective mixing. The prepared raw powder mixture was dried in a rotary evaporator to remove ethanol and then placed in a high-temperature furnace for calcination at 850^°^C for 4 h. The calcined powder mixture was subsequently ball-milled for 24 h to obtain an optimal particle size distribution for thick film preparation via AD.

### Fabrication of PZT-95/5 Thick films by AD

PZT-95/5 ceramic thick films were fabricated on Pt/Ti/SiO_2_/Si substrates via AD, as shown in Fig. S1 in Supporting Information (SI). The PZT-95/5 powder with an average particle size of ~ 1 μm (Fig. S2 in SI) was deposited on the substrate to form thick films via AD under a maintained vacuum level of 0.35 torr. A *De Laval*-type nozzle with a rectangular orifice with the dimensions of 0.5 mm × 10 mm was used in the deposition process. A mass flow controller was used to maintain the gas flow rate at 12 L/min, with medical-grade dry air serving as the carrier gas for transporting the active material powder through the supply line. The substrates were moved repetitively in the XY direction at a controlled speed of 1 mm/s to obtain 4-µm-thick films. The thick films were annealed at 600^°^C for 1 h, with the rates of temperature increase and cooling maintained at 1^°^C/min and 0.5^°^C/min, respectively [11]. The structural, morphological, and electrical properties of the prepared thick films were characterized to determine their functional characteristics.

### Characterization of PZT-95/5 thick films

The structural properties of the prepared PZT-95/5 powder and thick films were characterized using X-ray diffraction (XRD; XpertPro, Malvern Panalytical Ltd., Netherlands). The vibrational spectra of the thick films were evaluated using Raman spectroscopy (Nasoscope system 220 C, Korea). The surface morphological and cross-sectional micrographs of the prepared particles and films were captured using scanning electron microscopy (SEM; JIB-4700 F Multibeam system, JEOL, Japan). The focused ion beam (FIB) process (Helios 5 CX, Thermo Fischer Scientific, USA) was employed to slice the AD-prepared PZT-95/5 thick films into cross sections to analyze their grain structures at high magnification. Energy-dispersive X-ray spectroscopy (EDS) mapping and scanning transmission microscopy (STEM; Talos F200i, Thermo Fischer Scientific, USA) were also employed. To characterize the bulk material properties, the prepared powder was compacted into a disk by mixing it with a polyvinyl alcohol binder and sintering the mixture at 1250 °C for 2 h. To characterize the electrical properties, such as dielectric and FE characteristics, a metal–insulator–metal (MIM) capacitor device was fabricated by depositing a 5-mm-diameter Pt top electrode on the prepared thick film through a metal shadow mask using a DC sputtering system (Cressington Scientific Instruments, UK). A heat-treated silver electrode was used on bulk ceramic pellets to characterize the electrical properties. An impedance analyzer (Keysight E4990A, USA) was used to measure the dielectric properties in the frequency range of 100 Hz–1 MHz under an applied oscillation voltage of 0.5 V with the temperature varying from RT to 325^°^C. The FE properties of the bulk and thick film samples were characterized using a FE tester (Precision LC-II, Radiant Technologies, USA). Piezo force microscopy (PFM) was employed to characterize the amplitude and phase images of the prepared thick films under an applied bias of 1.5 V (Park Systems, Korea). The unipolar loops were measured to characterize the recoverable energy density and energy efficiency of the MIM-structured PZT-95/5 bulk ceramics and thick films. The stability of the thick films was further tested through a fatigue test for up to 10^7^ cycles and at temperatures up to 140^°^C. A custom-designed high-speed switching circuit was used to measure the time-dependent charge/discharge profiles of the PZT-95/5 thick film capacitors under a practical DC electric field of 0.45 MV/cm (180 V). The discharged profile was measured under a 1-kΩ load resistance using an oscilloscope (WaveSurfer 44Xs-A, Teledyne LeCroy Corp., USA).

## Results & discussion

**Fig. 1:**
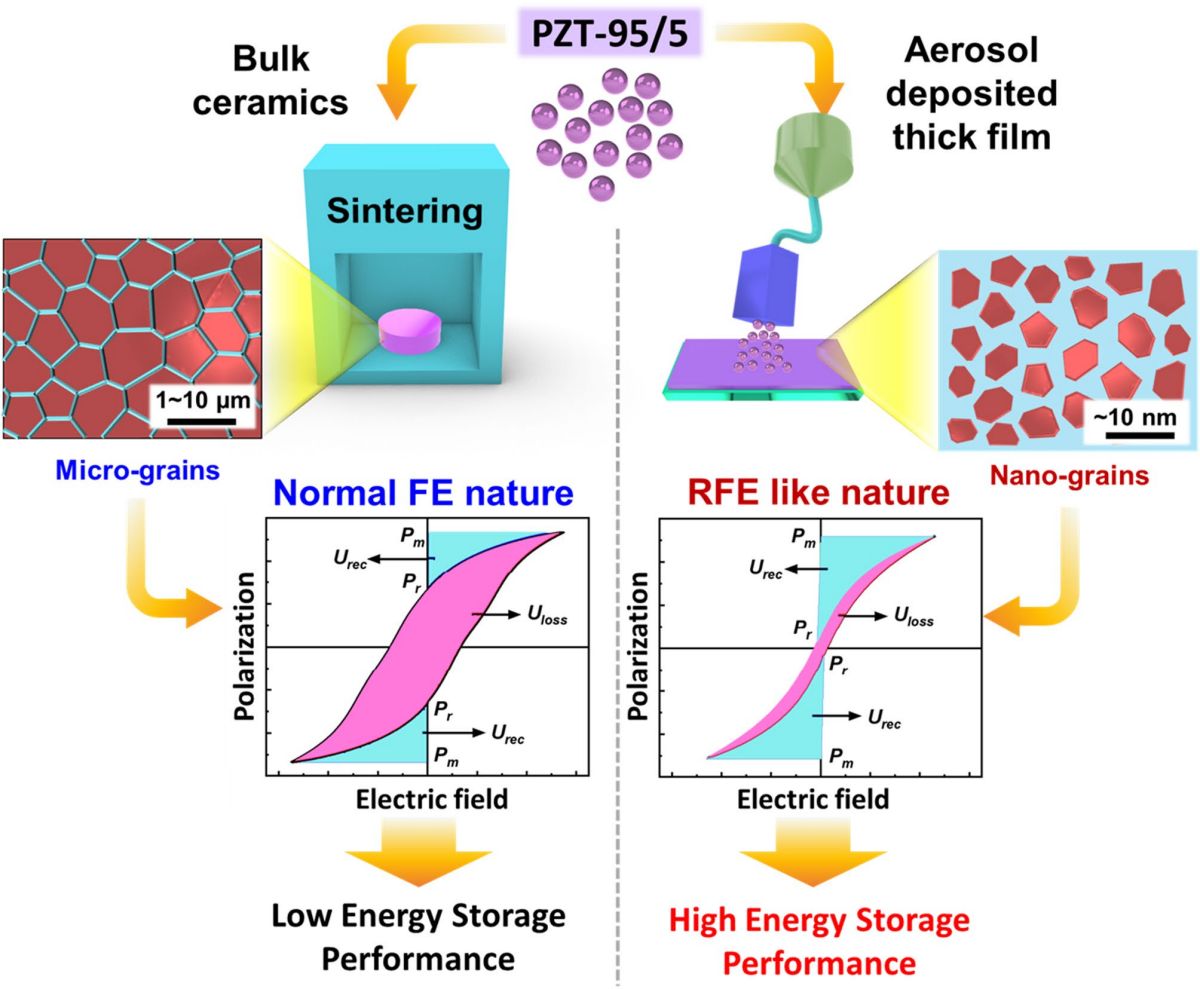
Schematic of PZT-95/5 ceramic powder conversion, either into micrograins via conventional sintering for bulk ceramics or nanograins surrounded by an amorphous matrix via AD. The PZT-95/5 bulk ceramic sample exhibits normal FE characteristics with significant hysteresis due to micrograin formation, whereas the PZT-95/5 thick film exhibits RFE-like characteristics with a slim hysteresis loop due to nanograin formation. This difference is represented by the change in their polarization–electric field (P-E) hysteresis loops. Here, *P*_*m*_, *P*_*r*_, *U*_*rec*_, and *U*_*loss*_ denote saturation polarization, remnant polarization, recoverable energy density, and energy loss, respectively

Figure 1 shows a schematic comparing the present AD-based thick film preparation and the conventional bulk ceramic preparation, along with the resultant effects on the FE hysteresis loops. Pellet formation and subsequent densification via high-temperature sintering resulted in the formation of large and irregularly shaped grains. This resulted in normal FE hysteresis loops with low *E*_*DBS*_ and *P*_*m*,_ which are generally unsuitable for energy storage. Bulk ceramics exhibit typical large FE hysteresis loops because of microdomain formation, and domain alignment occurs under an applied external electric field, resulting in a high *P*_r_, even under a zero electric field. In contrast, following the methodology of our previous study, the present study employed AD, which resulted in thick film formation. This formation yielded an optimized nanograin composite structure exhibiting RFE characteristics with a slim hysteresis loop and manifold increments in *E*_*DBS*_ and *P*_*m*_, indicating superior capacitive characteristics [8, 11]. The high kinetic energy of the AD process fractures ceramic microparticles into nanograins, which are then dispersed throughout the nonpolar amorphous matrix.

The nanograins, which are nearly identical in terms of domain size, form a flux-closure structure in the nonpolar amorphous matrix. This structure reduces the energy barrier for domain switching. Consequently, most nanodomains can switch back to their original random state under a zero electric field, resulting in low *P*_*r*_ [11]. The FE nature of nanograins and the nonpolar nature of the amorphous structure synergistically induce the RFE-like characteristics of the prepared thick film. The composite thick film sample exhibits a slim hysteresis loop, resulting from nanoscale domain formation in the nanograins and their random orientation in the nonpolar/amorphous matrix. This structure enables a high *E*_*DBS*_. Furthermore, the delayed saturation polarization results in RFE hysteresis loops, which collectively result in enhanced energy storage characteristics [11, 13].

**Fig. 2:**
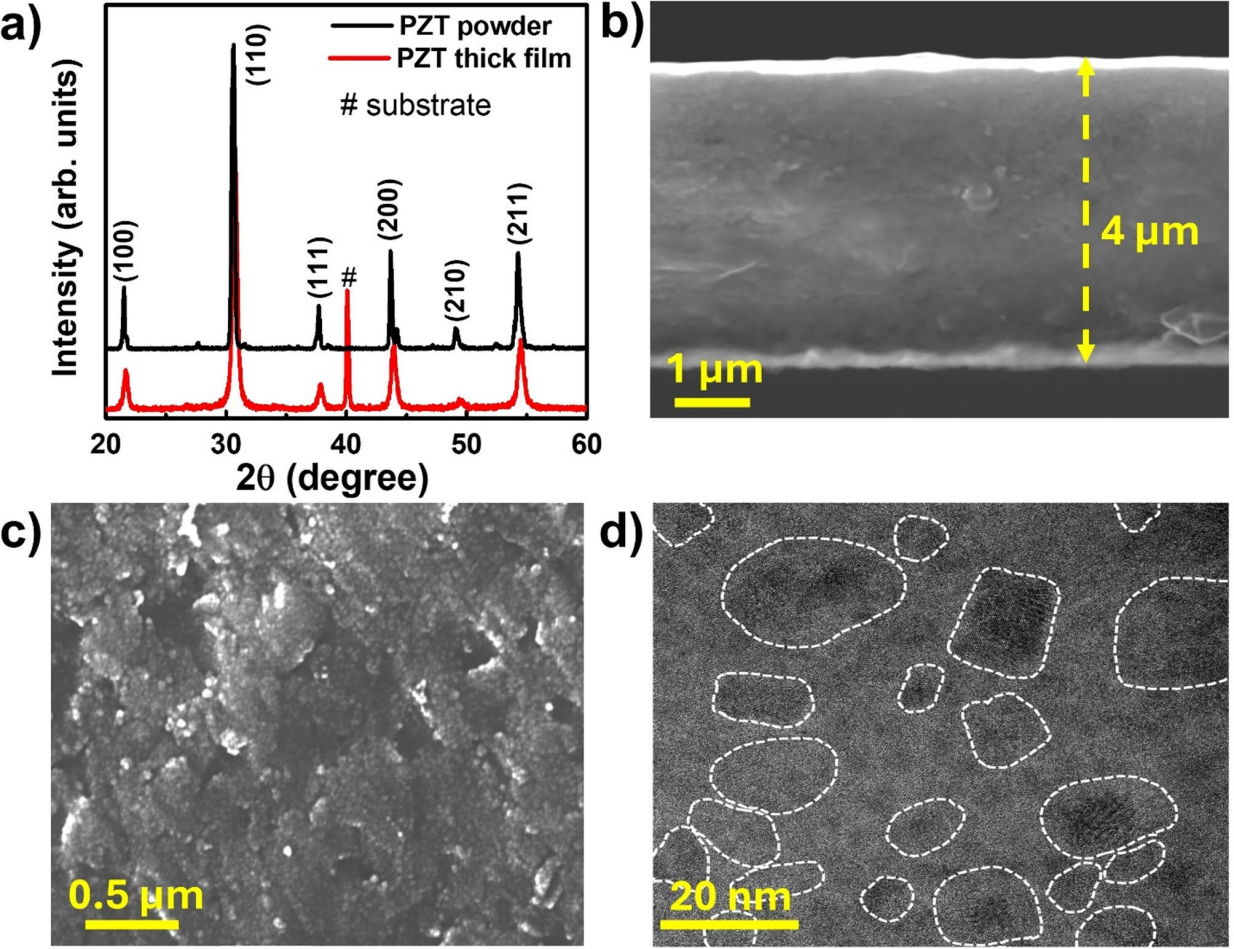
Structural and morphological analyses of PZT-95/5 powder and thick film. (**a**) XRD patterns of solid-state prepared, calcined PZT powder and annealed PZT thick film. (**b**) Cross-sectional view of AD-prepared PZT-95/5 thick film, revealing highly dense film formation with even thickness of ~ 4 μm. (**c**) Morphological surface view of PZT-95/5 thick film, displaying its dense surface with slight roughness. (**d**) HRTEM images of cross-section of annealed PZT-95/5 thick film, showing a nanograin formation with various interplanar spacings, formed by high kinetic energy during AD. (The white dashed lines indicate the formed nanograins.)

The crystal phases of the synthesized PZT-95/5 powder and thick films were analyzed using XRD. The results are presented in Fig. 2a. The PZT-95/5 system generally exists between an AFE orthorhombic phase and a FE rhombohedral phase at RT. The phase configuration depends on the domain size and the volume fraction of each phase in the prepared system [40, 44]. In this study, the PZT-95/5 powder for AD-based film deposition exhibits high intensity peaks with low full-width half-maximum (FWHM), and peak splitting is also observed at approximately 44º. A similar XRD pattern has been reported for Zr-dominated bulk and film samples, which is attributed to tetragonal distortion in AFE orthorhombic lattices, indicating the existence of FE and AFE phases [40, 44, 45]. The annealed thick films exhibit a similar pattern consisting of major peaks with enlarged FWHM, indicating a pseudocubic structure of nanocrystallites without any preferred orientation. The XRD results are further discussed in supporting note 3 and Fig. S3 in SI. The as-deposited films exhibited vibrational Raman bands at approximately 227, 330, 533, and 830 cm^− 1^ with enlarged FWHM, demonstrating their amorphous nature. After annealing, the intensity of the Raman modes was enhanced, and the FWHM was reduced to a level similar to the XRD results. The detailed Raman analysis is provided in Fig. S4 in SI.

The morphological microstructures of the prepared PZT-95/5 powder and thick films were analyzed using SEM. The micrographs are shown in Fig. 2b and c. Figure 2b shows the cross-sectional view of the annealed film, showing highly dense microstructure formation with ~ 4 μm thickness during AD. The cross-sectional and surface views of the annealed thick film show densely deposited PZT-95/5 particles. Fig. S5 shows the SEM-EDS mappings of thick films. The elemental maps indicate homogeneous distribution of all elements throughout the thick film surface. The highly dense microstructure of the thick film comprises nanograins and an amorphous structure with a high grain boundary density, resulting in enhanced *E*_*DBS*_, which is vital for high-energy density performance. This result is supported by the high-resolution transmission electron microscopy (HRTEM) characterization of the annealed PZT-95/5 thick film, which is sliced by the FIB process. The high-resolution view of the cross-sectionally sliced film surface is shown in Fig. 2d, showing several polar nanograins in a nonpolar matrix composed of PZT-95/5. The film exhibited nanograins with sizes ranging from ~ 8 to ~ 28 nm with an average size of ~ 15 nm. High-kinetic energy collisions during the AD process fractured the PZT-95/5 microparticles, resulting in the formation of smaller crystallites in the thick film (Fig. 2d and Fig. S6). The STEM-EDS mapping of the PZT-95/5 film is shown in Fig. S7.

**Fig. 3:**
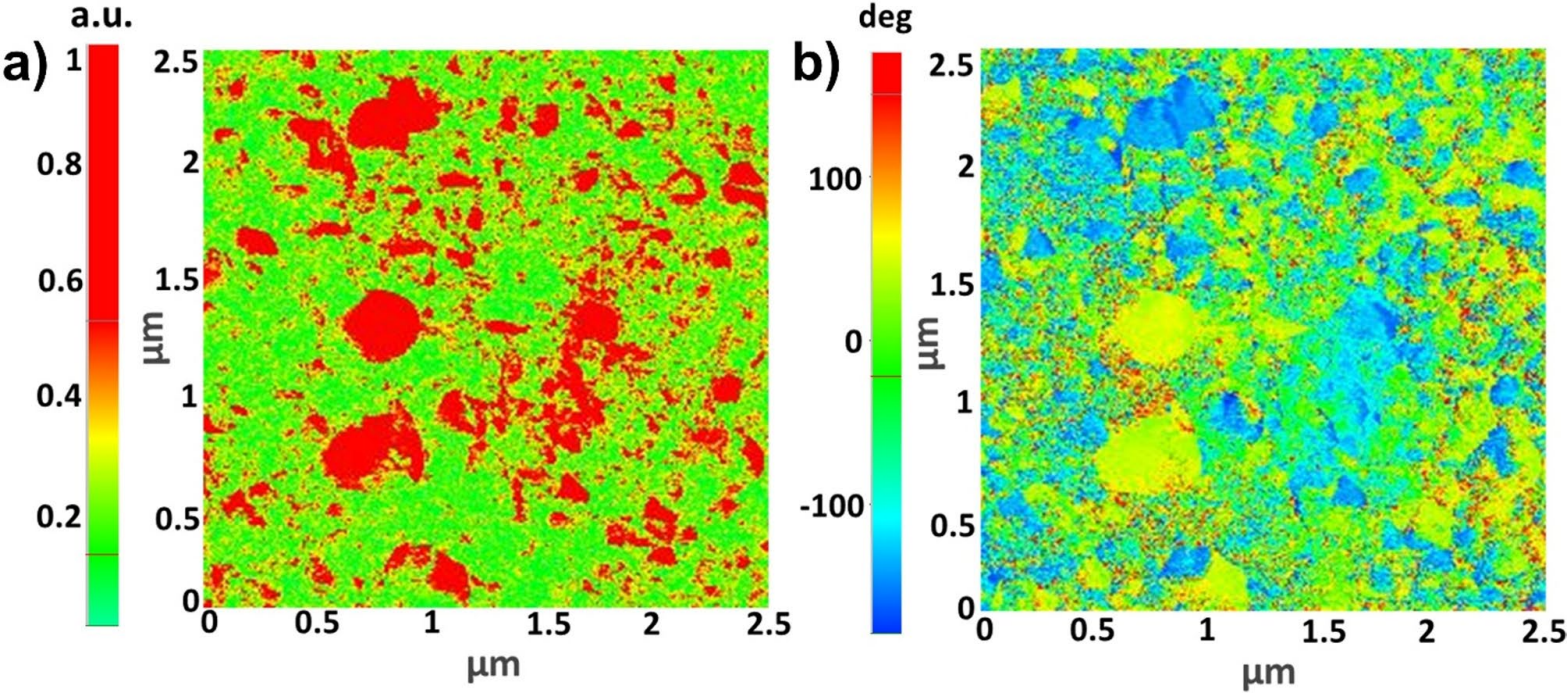
PFM analysis of AD-prepared PZT-95/5 thick film at scanning area of 2.5 × 2.5 µm^2^. (**a**) PFM amplitude and (**b**) phase images of annealed AD-prepared PZT-95/5 thick film

The AD-prepared PZT-95/5 film was characterized by PFM to investigate its topography, amplitude, and phase. The results (Fig. 3 and Fig. S8) provide evidence that the AD process mechanically fractures the micrograins, transitioning them into PZT nanograins surrounded by an amorphous nonpolar matrix. The topography image in Fig. S8 shows that the film exhibits a slightly higher roughness resulting from high-kinetic energy particle–substrate collisions in vacuum. The absence of physical damage to the film surface indicates successful film formation via the AD process. The reddish-yellow patches in the amplitude image of Fig. 3a represent the formed nanograins with different polarization strengths, which are surrounded by the continuous distribution of an amorphous nonpolar phase. This ensures the formation of a film composed of FE nanograins embedded in a nonpolar amorphous matrix. Figure 3b shows the phase image of the AD-prepared PZT-95/5 film, displaying a random polarization orientation, indicating the presence of complex domains. These domains cause delayed saturation polarization, resulting in high *P*_*m*_ and superior energy storage characteristics.

**Fig. 4:**
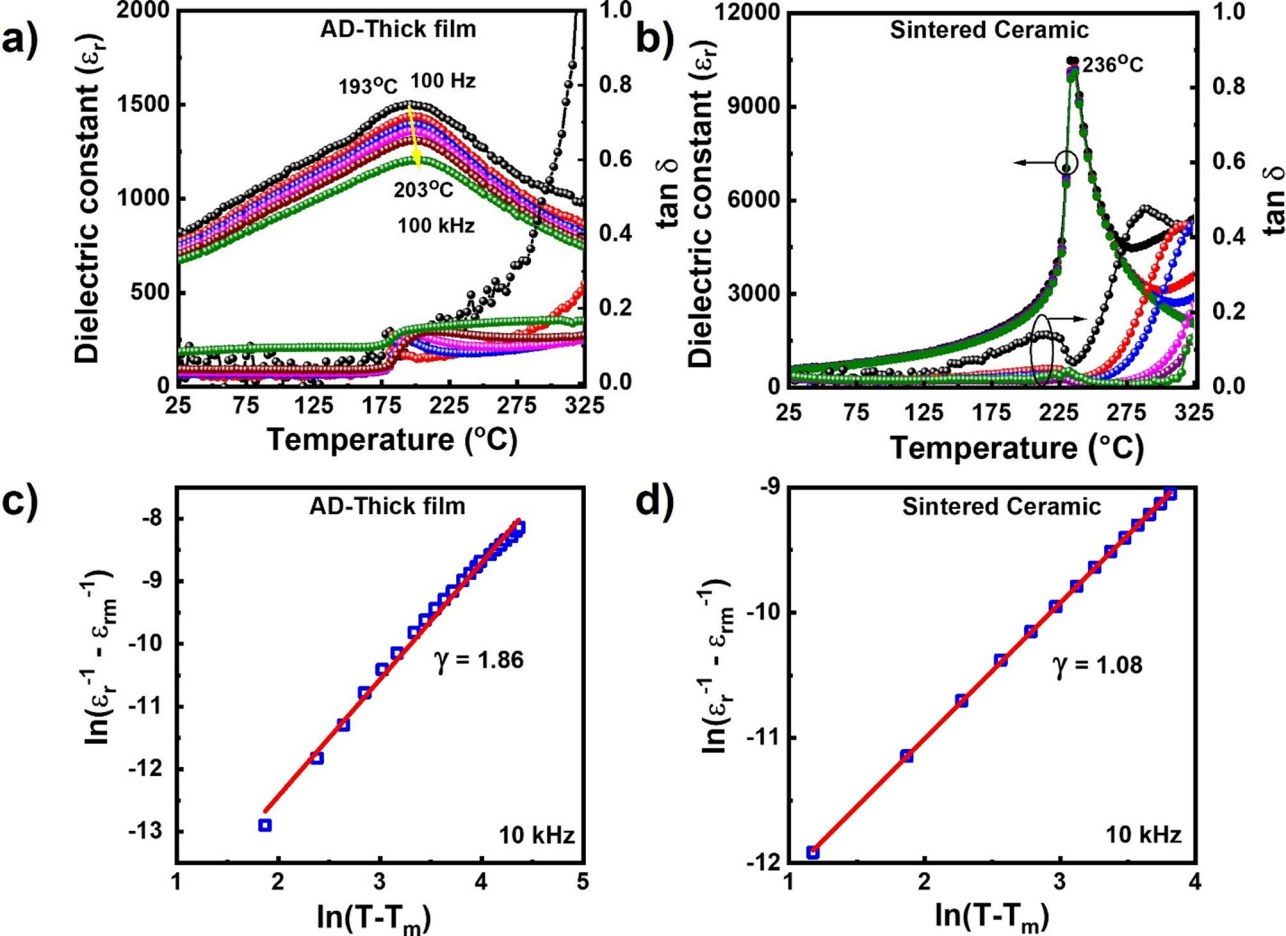
Dielectric characterizations of AD-prepared PZT-95/5 thick films and sintered bulk ceramic samples. Temperature-dependent dielectric properties at various temperatures ranging from RT to 325 °C (dielectric constant (*ε*_*r*_) and loss tangent (*tanδ*)) of **(a)** PZT-95/5 thick film and **(b)** PZT-95/5 bulk ceramics at different measurement frequencies, showing the dispersion nature in *T*_*C*_ for thick films, whereas bulk ceramics exhibit a sharp *T*_*C*_ at 236 °C. Plots of ln (1/$\:\epsilon\:$_r_ − 1/$\:\epsilon\:$_m_) vs. ln (T - T_m_) for **(c)** PZT-95/5 thick film and **(d)** PZT-95/5 bulk ceramics measured at 10 kHz to identify the dielectric diffuseness factor (*γ*). The thick films and bulk ceramics exhibit *γ* values of 1.86 and 1.08, respectively

The dielectric constant (*ε*_*r*_) and loss tangent (*tanδ*) of the AD-prepared PZT-95/5 thick films at temperatures ranging from RT to 325 °C and frequencies ranging from 100 Hz to 100 kHz were measured. The results are presented in Fig. 4a. The AD-prepared thick film exhibits a dielectric constant value of ~ 776 at RT and 1 kHz. A phase transition is observed at 193 °C and 100 Hz, which moves to a higher transition point at 203 °C and 100 kHz, indicating the relaxor-like dielectric dispersion behavior of the PZT-95/5 thick films. In contrast, the dielectric loss remains below 0.2 at temperatures ranging from RT to 240^°^C. The lower transition temperature in the AD-prepared PZT-95/5 films than in bulk ceramics (236^°^C) can be attributed to different factors, including surface and size effects and internal stress resulting from the AD process. This is further discussed in supporting note 9 in SI [46–48].

For comparison, the dielectric properties of the bulk ceramics were evaluated at various temperatures and measurement frequencies ranging from 1 kHz to 1 MHz (Fig. 4b). The bulk ceramics exhibited a dielectric constant of ~ 587 at RT and 1 kHz; the phase transition from FE to paraelectric was observed at ~ 236^°^C without any dispersion at various frequencies, indicating the good FE nature of the material. Furthermore, the dielectric loss follows a similar trend. The dispersion phenomena in the thick film and bulk samples were further evaluated. The results are presented in Fig. 4c and d. The diffuseness coefficients of the PZT-95/5 bulk and thick film samples were determined from the slopes of the ln (1/ε_r_ − 1/ε_m_) vs. ln (T - T_m_) lines at 10 kHz (Fig. 4c and d). The bulk sample exhibited a coefficient of 1.08, whereas the thick film sample exhibited an increased coefficient of 1.86, confirming the enhanced RFE properties of the AD-prepared film. The mechanically tailored PNR formation in the thick film sample induces RFE properties rather than typical heterogeneity-induced relaxor characteristics. The long-range ordered microdomains of normal FE materials were converted into dense and short-range ordered nanodomains by the high kinetic energy of AD during film fabrication. This resulted in the diffusive dielectric properties observed in the PZT-95/5 thick films [11].

**Fig. 5:**
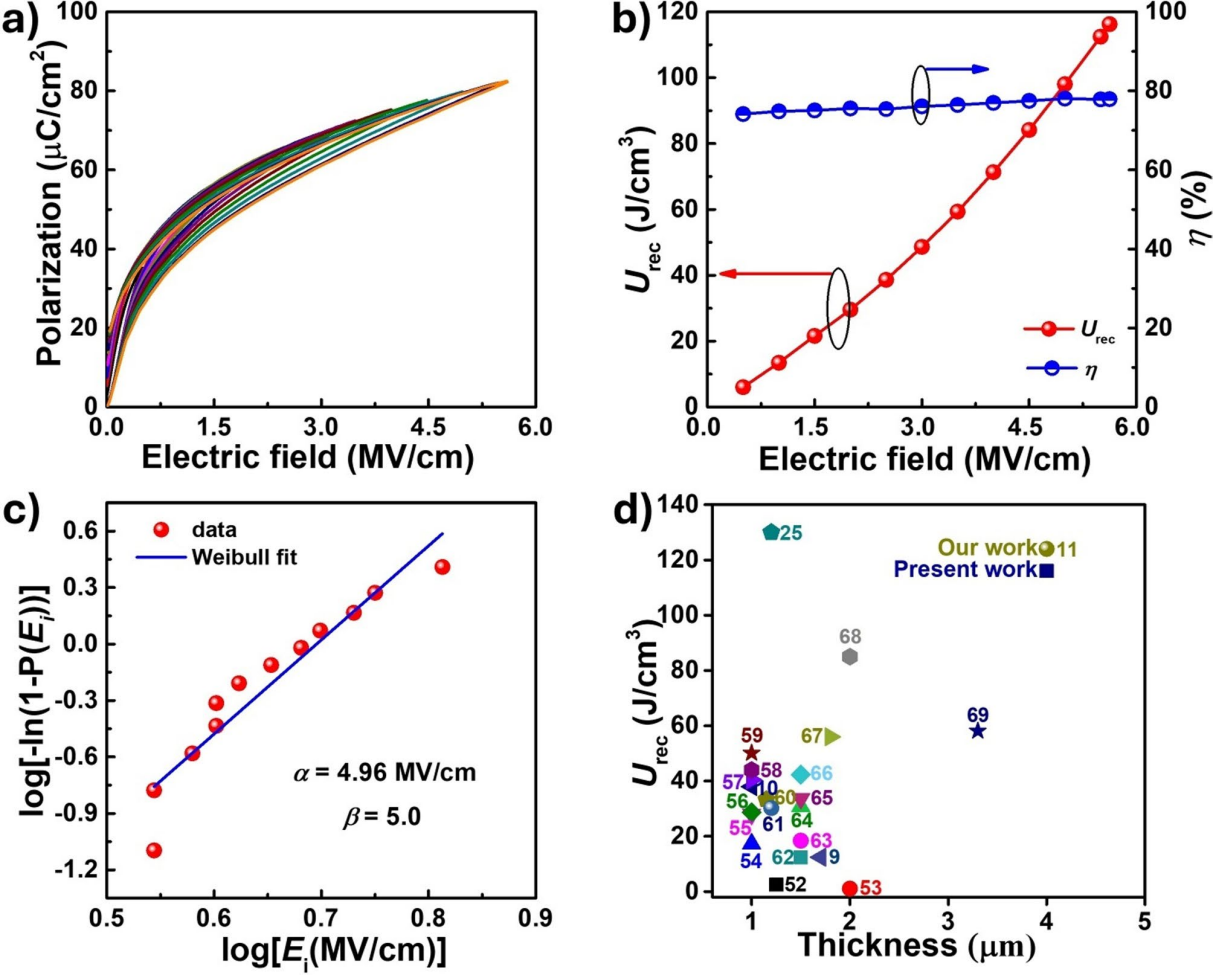
FE characterizations and energy storage analysis of PZT-95/5 bulk ceramic and thick film samples. **(a)** FE unipolar loops of PZT-95/5 thick film, measured under increasing applied electric fields up to the breakdown strength. These are used to determine energy storage properties. **(b)** Calculated recoverable energy density (*U*_*rec*_) and efficiency (*η*) of PZT-95/5 thick film under various applied electric fields. **(c)** Weibull distribution for evaluating the most probable breakdown field of AD-prepared PZT-95/5 thick film. **(d)** Comparison of recoverable energy densities of recently reported state-of-the-art high-performance dielectric capacitors with micron-scale thickness

The mechanically tailored RFE-like behavior observed in the samples was further analyzed by conducting FE *P-E* loops studies on the prepared thick film (Fig. 5) and the bulk sample (Fig. S9a in SI). The AD-prepared thick films exhibited RFE-like slim hysteresis loops. The mechanically induced transformation from micrograins into nanograins by AD resulted in the formation of nanopolar grains into an amorphous structure. This enables the domains to easily return to their original random orientation upon removal of the electric field, resulting in low *P*_*r*_ and a low coercive field (*E*_*c*_) with the slim hysteresis loops. The large *P*_*m*_ value of the PZT-95/5 thick film can be attributed to the field-induced growth of nanograins/domains. In the case of normal FE materials, the majority of the domains remain in order, resulting in a high *P*_*r*_ even upon the removal of the electric field due to the little back-switching of macroscopic domains. In addition, the amorphous matrix surrounding these polar nanograins and additional grain boundaries in the thick film can act as a depletion space charge layer, which can limit charge carrier movement. This results in a low leakage current for the prepared thick films upon the removal of the external electric field, thereby improving the *E*_*DBS*_ [49, 50]. The nanograin size and its ratio with the matrix are critical factors for the enhanced FE polarization and *E*_*DBS*_, which are expected to yield good *U*_*rec*_ and *η* [11].

To determine the energy storage properties of the PZT-95/5 thick films, unipolar *P-E* loop characterization was performed. The results are presented in Fig. 5a. The device exhibited a large *E*_*DBS*_ value of ~ 5.6 MV/cm and *P*_*max*_ of 80 µC/cm^2^. The thick film samples exhibited a manifold increase in *E*_*DBS*_, demonstrating the advantage of using AD to achieve high-energy density capacitors. The *U*_rec_ and *η* values of the sample under various electric fields are presented in Fig. 5b. The *U*_*rec*_ value linearly increased with the electric field, reaching a maximum of 116 J/cm^3^ at a high *E*_*DBS*_ value of 5.6 MV/cm. Furthermore, the device efficiency remained constant at 78% across the entire range of applied fields, demonstrating the suitability of the device for practical applications.

**Fig. 6:**
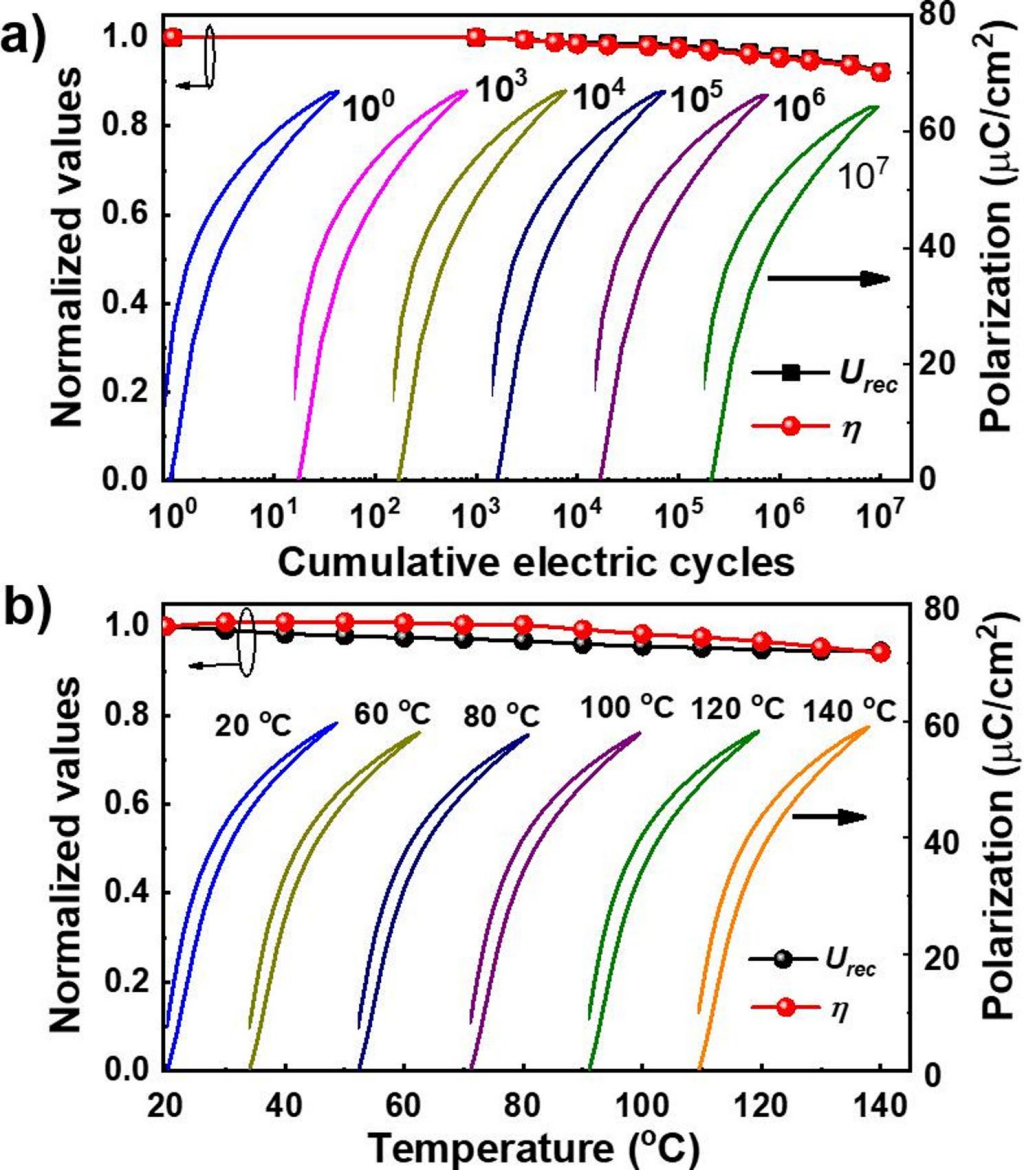
Reliability performance characterization of AD-prepared PZT-95/5 thick film capacitor. **(a)** Fatigue characterization of PZT-95/5 thick film capacitor in terms of unipolar *P-E* hysteresis, recoverable energy density (*U*_*rec*_), and efficiency (*η*) for 10^7^ electric cycles under applied electric field. **(b)** Thermal stability characterization of PZT-95/5 thick film capacitor in terms of its polarization, *U*_*rec*_, and *η* from RT to 140 °C under an applied electric field of 1 MV/cm (400 V)

The robustness of the AD-prepared PZT-95/5 thick film capacitors under an electric field was evaluated in terms of their *E*_*DBS*_ value using the Weibull (WB) distribution function. The results are presented in Fig. 5c [11, 51]. The Weibull scale parameter (α), representing the field for a 63.2% breakdown probability, and the shape parameter (β), indicating the distribution slope, were determined to be 4.96 MV/cm and 5.0, respectively. The smaller *β* value indicates the non-uniform distribution of nanograins in terms of the grain size in the amorphous matrix of the AD-prepared PZT-95/5 films. Under this characteristic electric field of 4.96 MV/cm, the calculated recoverable energy density (*U*_*rec*_) and efficiency (*η*) are 98.02 J/cm^3^ and 78.06%, respectively (Fig. S9b). Furthermore, the high *U*_*rec*_ performance of the fabricated capacitors was compared with that of recently reported thick film capacitors on various substrates with micron-scale film thickness (Fig. 5d and Table S1). The AD-prepared PZT-95/5 capacitor developed in this study significantly outperformed the recently reported capacitors [9–11, 25, 52–69]. This is primarily because of the mechanically tailored RFE-like nature of the AD-prepared PZT-95/5 film, resulting in low remanent polarization and a significantly enhanced breakdown field, enabling high energy storage performance.

In addition to its high energy storage performance, the stability of the AD-prepared PZT-95/5 capacitor under fatigue cycling and temperatures is critical for practical applications. As shown in Fig. 6a, the AD-prepared PZT-95/5 capacitor device underwent fatigue cyclic testing for 10^7^ cycles under an applied field of 1.63 MV/cm (650 V). The device performance parameters, namely *U*_rec_ and *η*, exhibited minimal degradation, retaining 92% of their initial values after 10^7^ cycles, demonstrating excellent fatigue endurance. Considering that capacitors are used under harsh conditions, stable performance at various temperatures is another important factor for practical applications. The AD-prepared PZT-95/5 capacitor device was tested at various temperatures ranging from RT to 140^°^C under an applied electric field of 1 MV/cm (400 V). The results are presented in Fig. 6b. The capacitor comprising the AD-prepared PZT-95/5 ceramic thick film exhibited a slim hysteresis loop across the entire temperature range, demonstrating its good temperature stability. The negligible changes in the *P-E* hysteresis loops with increasing temperature demonstrate the exceptional insulation properties of the AD PZT-95/5 thick films even at elevated temperatures. The mere 6% variations in the *U*_rec_ and *η* of the PZT-95/5 capacitor device across the entire temperature range demonstrate its reliable performance and suitability for practical applications.

**Fig. 7:**
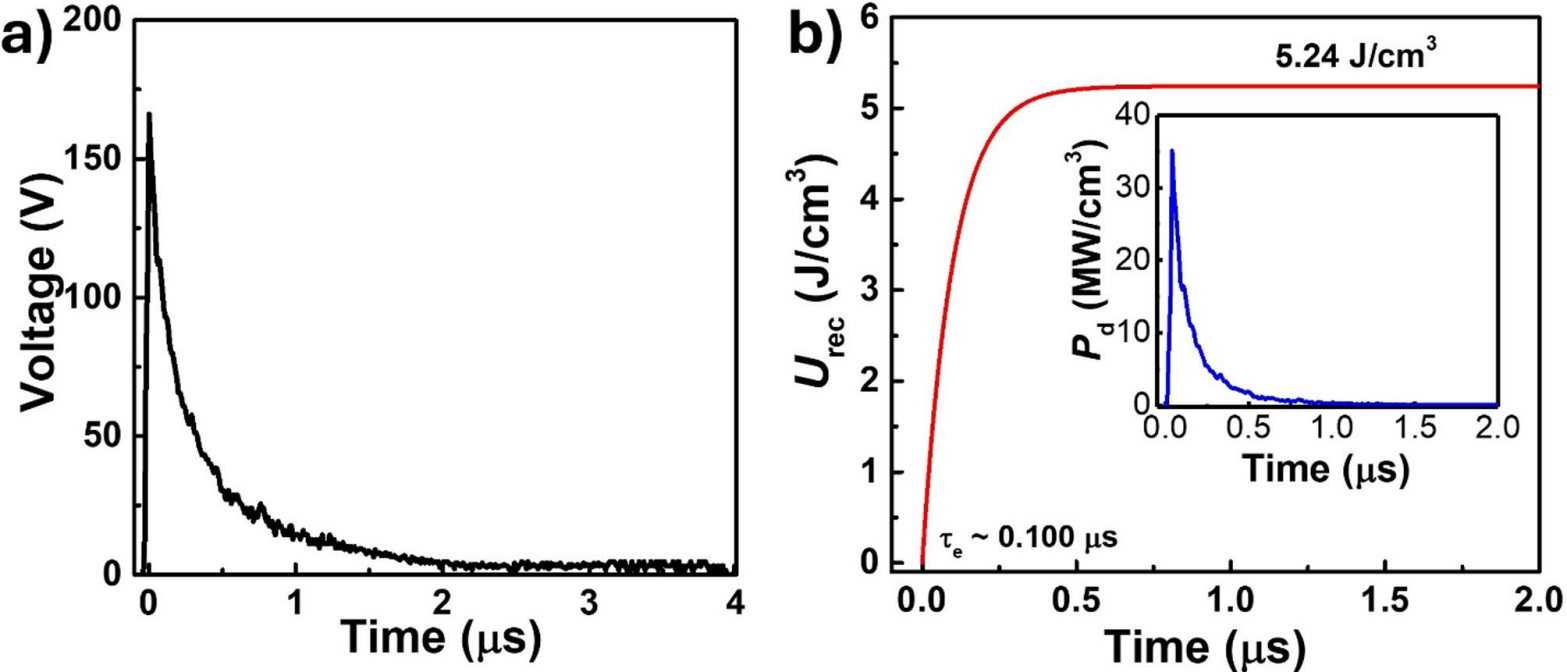
Charge–discharge performance of AD-prepared PZT-95/5 thick film capacitor. **(a)** Time-dependent charge–discharge voltage profile under 180 V and 1 kΩ resistance. **(b)** *U*_rec_ and τ_0.90_ of PZT-95/5 thick film capacitor. The inset figure shows the instantaneous power density estimated from the time-dependent voltage profile

The charge–discharge performance of the AD-prepared PZT-95/5 thick film capacitor device was evaluated under static conditions in terms of its *U*_rec_ and power density (*P*_*d*_). The charge–discharge cycle measurement setup is shown in Fig. S10. The AD-prepared PZT-95/5 thick film capacitor was charged under a DC electric field of 0.45 MV/cm (180 V), and the stored energy of the capacitor was discharged through a commercial load resistance of 1 kΩ. The resulting time-dependent charge–discharge voltage profiles were recorded using an oscilloscope (Fig. 7a). The *U*_rec_, time required for discharging 90% of the stored energy (τ_0.9_), and power density (*P*_*d*_) were measured (Fig. 7b). The discharge energy density was calculated as follows:

1 $${U_{{\rm{rec}}}} = \smallint _0^tV\left( t \right)I\left( t \right)dt = \frac{{\tau \:V_0^2}}{{2vR}}\left[ {1 - {e^{ - \frac{{2t}}{{\tau \:}}}}} \right],$$

where V(*t*) denotes the voltage at time *t*, *I*(*t*) denotes the current at time *t*,* R* represents the load resistance, τ represents the relaxation time, and *v* represents the capacitor film volume. The instantaneous power density of the AD-prepared PZT-95/5 capacitor device was calculated using the equation *P*_*d*_ = $\:\frac{{\left(V\left(t\right)\right)}^{2}}{R}$. The calculated discharged energy density (*U*_rec_) and power density (*P*_d_) of the capacitor are shown in Fig. 7b. Under an applied voltage of 180 V_DC_, the maximum *U*_rec_ was 5.24 J/cm^3^ with a τ_0.9_ value of 230 ns. The instantaneous peak power density of 35 MW/cm^3^ of the fabricated AD-prepared PZT-95/5 capacitor demonstrates its potential for pulsed power applications.

Overall, these results demonstrate that AD-prepared PZT-95/5 capacitors are suitable for practical energy storage applications because of their high recoverable energy density, exceptional fatigue endurance, and excellent temperature stability. They are also suitable for pulsed power applications owing to their fast charge and discharge properties.

## Conclusion

Nanograin engineering applied to PZT-95/5 via AD transformed its normal FE nature into a RFE nature. This nanostructuring approach differs from the traditional composition modification effect and its associated complex process. The distribution of the formed nanograins surrounded by a nonpolar matrix induced the RFE-like properties, which are critical for achieving high energy storage properties. The AD-prepared thick films (~ 4 μm) exhibited a remarkable *E*_*DBS*_ value of ~ 5.6 MV/cm and a high recoverable energy density of 116 J/cm^3^. This performance is achieved by the formation of nanograins with nanodomains distributed across a nonpolar amorphous matrix. This matrix acts as a flux, reducing the charge barrier and facilitating easy domain switching. Furthermore, the high density of insulating grain boundaries hinders charge flow, resulting in a significantly large *E*_*DBS*_. Collectively, these effects enhance the energy storage characteristics. The PZT-95/5 capacitor exhibited reliable fatigue endurance up to 10^7^ cycles and good thermal stability up to 140^°^C, demonstrating its potential for practical applications. The presented simple modification strategy, which significantly enhances the energy storage properties, even in normal FE/AFE systems, holds significant promise for developing next-generation multifunctional smart devices.

## Supplementary Information

Below is the link to the electronic supplementary material.

Supplementary Material 1

## Data Availability

Data will be made available upon reasonable request.
