# Supplementary material for: Emergence of relaxor-like ferroelectric nature in nanograined Pb(Zr0.95Ti0.05)O3 ceramic thick films for energy storage applications
Source: Nano Converg. 2025 Sep 29;12:46. doi: 10.1186/s40580-025-00511-3 (PMC12480236; doi:10.1186/s40580-025-00511-3)
Supplement: Supplementary file 1 — Supplementary Material 1 [file 40580_2025_511_MOESM1_ESM.pdf]

Supporting Information

Emergence of relaxor-like ferroelectric nature in nanograined $Pb(Zr_{0.95}Ti_{0.05})O_3$ ceramic thick films for energy storage applications

Nirmal Prashanth Maria Joseph Raj[1], Hyunseok Song[2], Satyabrata Lenka[1], Geon-Tae Hwang[3], Dae-Yong Jeong[4], Mahesh Peddigari[1*], Jungho Ryu[2*]

[1] Department of Physics, Indian Institute of Technology Hyderabad, Kandi, 502284, Telangana, India

[2] School of Materials Science and Engineering, Yeungnam University, Daehak-ro, Gyeongsan-si, Gyeongsangbuk-do, Republic of Korea

[3] Department of Materials Science and Engineering, Pukyong National University, 45, Yongso-ro, Nam-Gu, Busan, Republic of Korea

[4] Program in Semiconductor Convergence, Department of Materials Science and Engineering, Inha University, Incheon, Republic of Korea

[*] Indicates the corresponding author

* Corresponding author: Mahesh Peddigari, Jungho Ryu

Tel.: +91-4023016726, +82-53-810-2474

E-mail address: mahesh.p@phy.iith.ac.in, jhryu@ynu.ac.kr

Supporting figures and notes

1. **Aerosol deposition:**

Fig.S1 presents the schematic representation of the AD equipment used in the thick film preparation. Under the vacuum condition, the high kinetic energy of the carrier gas flow transferring the micron sized PZT-95/5 particles from aerosol generation chamber to deposition chamber. The transferred particles collide on the substrate surface fractured into the composite of nanograins of PZT-95/5 and the highly disordered (amorphous) matrix. The applied mechanical energy controlled the nanograin size and distribution of them, while surrounded by the amorphous matrix in the microstructure. The post-annealing after AD at the 600ºC, helps in grain growth to form the densified thick film. The formed nanograins are dispersed in the non-polar amorphous matrix, that provides the advantage of reduction in the energy barrier for the domain switching, leads to the formation of RFE like characteristics with larger P_m and slim hysteresis loops, preferable for the energy storage applications as schematically presented in Fig.1.

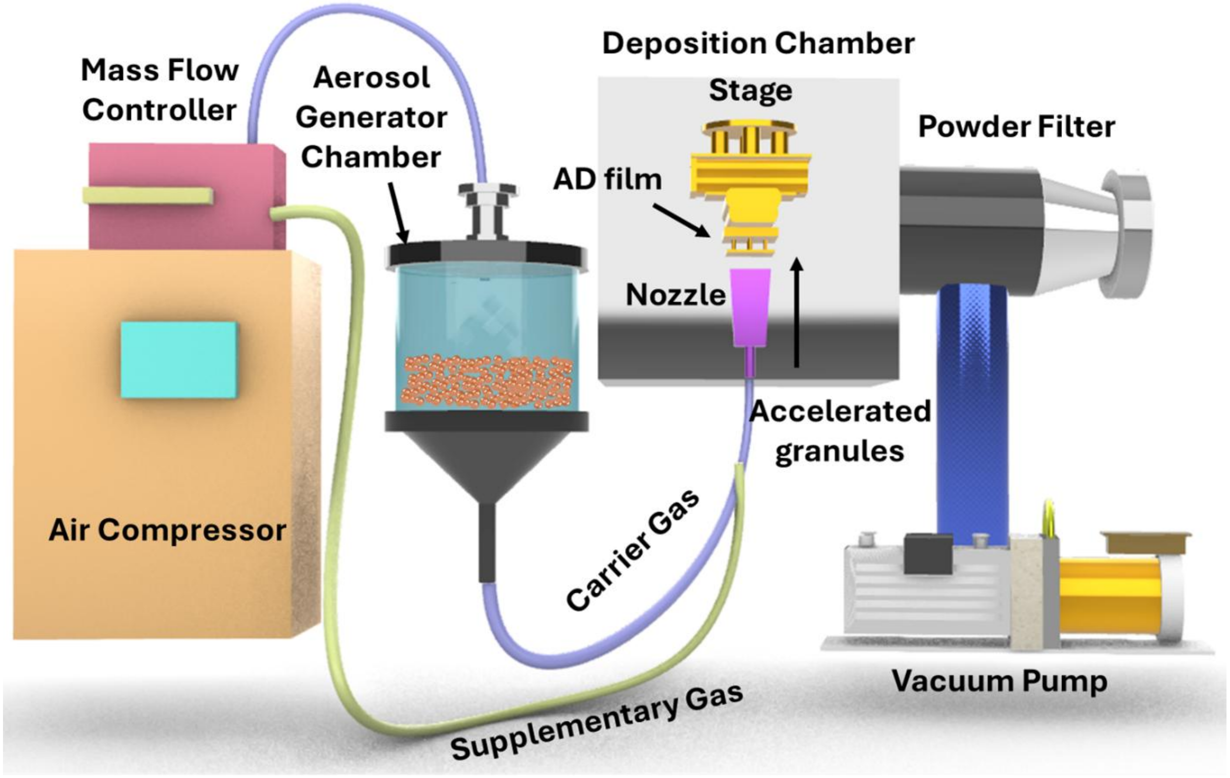

Figure S1. Schematics of aerosol deposition apparatus used to prepare the PZT-95/5 ceramic thick film [1]

2. SEM micrographs of PZT-95/5 particles:

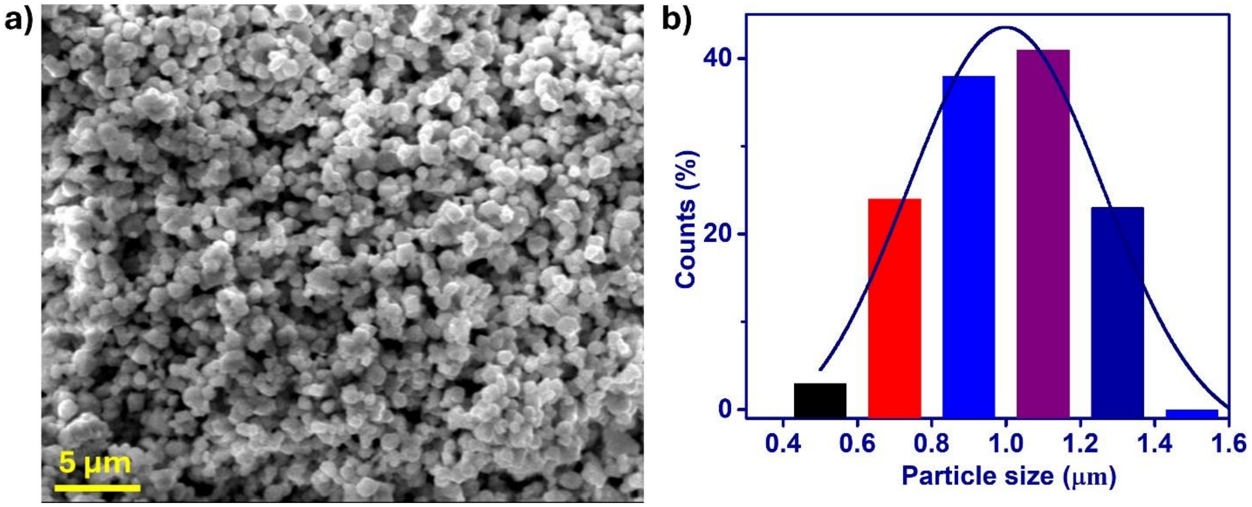

Figure S2. (a) SEM micrograph of the prepared bulk PZT-95/5 ceramic particles. **(b)** PZT-95/5 particles size distribution histogram determined from the SEM image.

Fig.S2a presents the SEM micrograph of PZT-95/5 ceramic particles synthesized via solid state reaction route and sequentially 24 hrs ball milled for AD process. The prepared calcined particles underwent ball milling to make them nearly uniform in particle size, and the attained final particles are in 1 μm size range, confirmed by the histogram presented in Fig.S2b, derived from SEM image using the ImageJ software.

3. **XRD patterns of PZT-95/5 powder and thick film:**

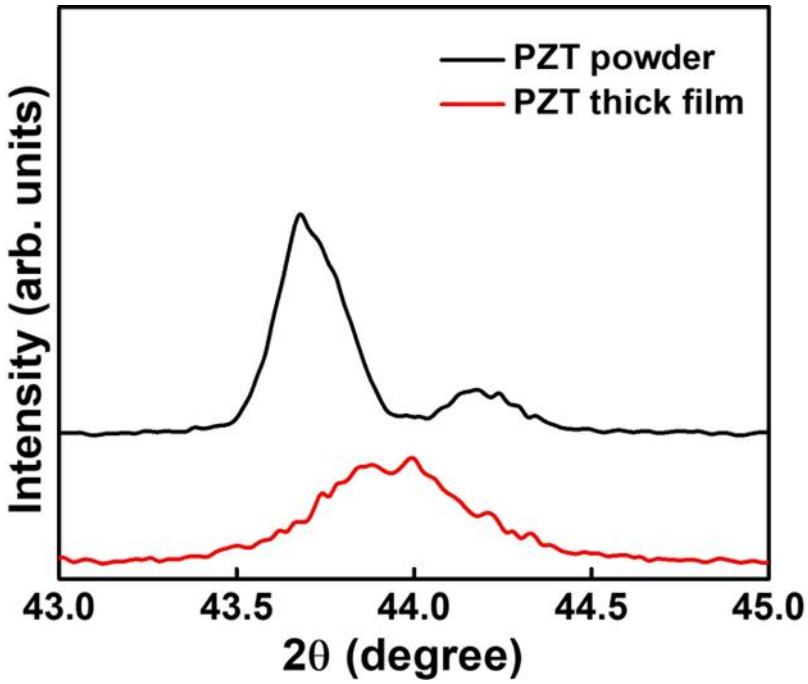

Figure S3. The magnified view of the XRD pattern of PZT powder and AD thick film around 2θ of 44°.

The XRD pattern of the calcined powder for AD process exhibits high intensity peaks with lower FWHM, indicative of its good crystalline nature. While the PZT thick film sample exhibits relatively lower intense peaks with higher FWHM denoting lower crystalline (crystallite size ~ 19.3 nm) than the powder (crystallite size ~ 30.9 nm) sample. The powder sample having the vicinity of orthorhombic and tetragonal phase exhibits the peak splitting around 45°. During thick film formation, through the Aerosol Deposition (AD) process, developed high kinetic energy fractures the particle into nanograins dispersed in the non-polar amorphous matrix, reasoning the formation of the lower crystalline with pseudocubic in nature. Fig.S3 presents the magnified view of the peaks around 44°, indicates that thick film samples don't have any peak splitting. The magnified view reassures that the powder sample did have better crystalline also have peak splitting, suggesting the existence of FE and AFE phases collectively, unlike the thick film. The similar kind of difference between the bulk and thick films samples have been well reported in the literature [2, 3].

4. Raman analysis:

In general, PZT 95/5 exhibits a temperature dependent phase with the unique spectrum that corresponds to the individual space groups. From high temperature to low temperature, the PZT 95/5 generally reveals the paraelectric cubic phase at high temperature of above T_c, to the high temperature ferroelectric phase (FE_{HT}) with Rhombohedral *R3m* space group, to low temperature ferroelectric phase (FE_{LT}) with the Rhombohedral *R3c* space group and finally shown antiferroelectric state with the *Pbam* space group in the order of $PE \rightarrow FE_{HT} \rightarrow FE_{LT} \rightarrow AFE$. However, based on the Raman spectrum performing experimental conditions (scanning speed, thermal rate) the PZT 95/5 exhibits the FE_{HT}, FE_{LT} and AFE phases within ~ 290 K and ~ 305 K temperature range. The microscopic Raman study for the as deposited and annealed thin film samples were performed under the excitation wavelength of 532 nm between the wavenumber of 120 cm^{-1} to 1000 cm^{-1}, and the observed results are presented in Fig.S4.

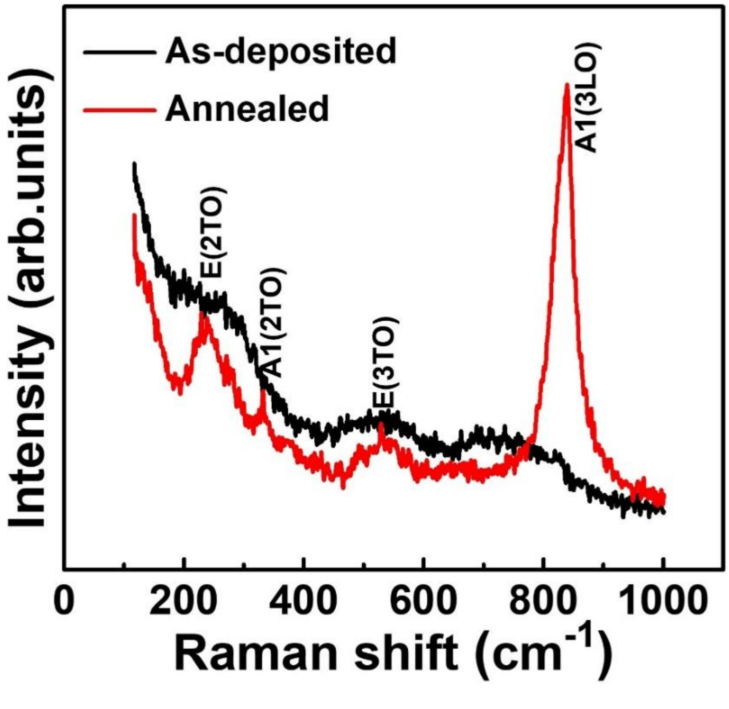

Figure S4. Raman spectra of the as deposited and annealed PZT-95/5 thick films.

The as deposited film exhibited the vibrational bands with broader FWHM indicates the amorphous nature of the film. After annealing at 600 ºC for an hour, the deposited nanograin composite thick film revealed the vibration modes around 227 cm^{-1}, 330 cm^{-1}, 533 cm^{-1} and dominant peak at 835 cm^{-1}. The vibration E(2TO) mode at 227 cm^{-1} assigned to the B-site vibration like Zr–O and Ti–O vibrations common to all three space groups. The unique vibration A_1(2TO) mode at 330 cm^{-1} suggestive of the FE_{LT} phase, while the 533 cm^{-1} vibration E(3TO) mode can be assigned to both the FE_{LT} and AFE phases [4-6]. The dominant, high intense, unique Raman vibration A_1(3LO) mode emerged at 835 cm^{-1} can be attributed to the higher substitution of the Zr content at Ti, representing the expansion of octahedron TiO_6 and ZrO_6 at the same location, and indictive of the relaxor phase characteristics arise in the sample [7-9].

5. SEM-EDS elemental mapping of the AD thick film:

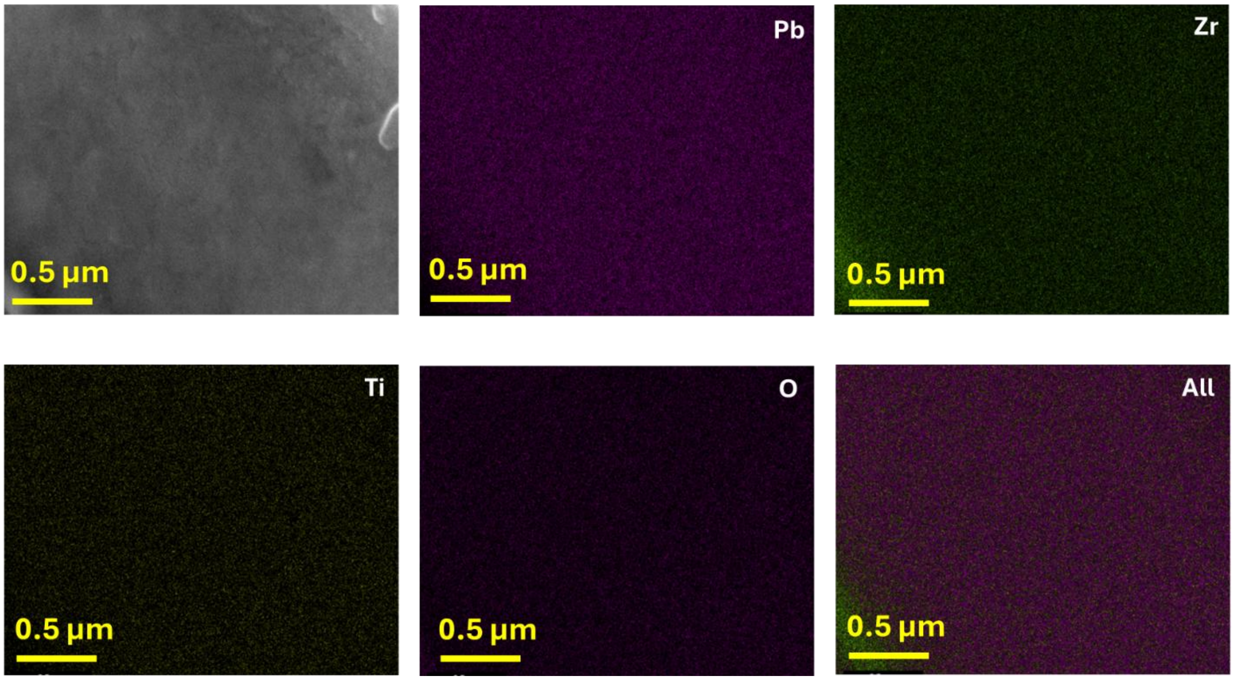

Figure S5. SEM-EDS elemental maps obtained from the annealed PZT-95/5 AD thick film.

Fig.S5 presents the Scanning Electron Microscopy (SEM)-Energy dispersive X-ray spectroscopy (EDS) maps of the elements Pb,Zr,Ti and O attained from the annealed AD thick film. The uniform color grading in the entire area of the prepared thick film reveals the homogeneous elemental distribution.

6. HR-TEM surface graphs of the AD thick film

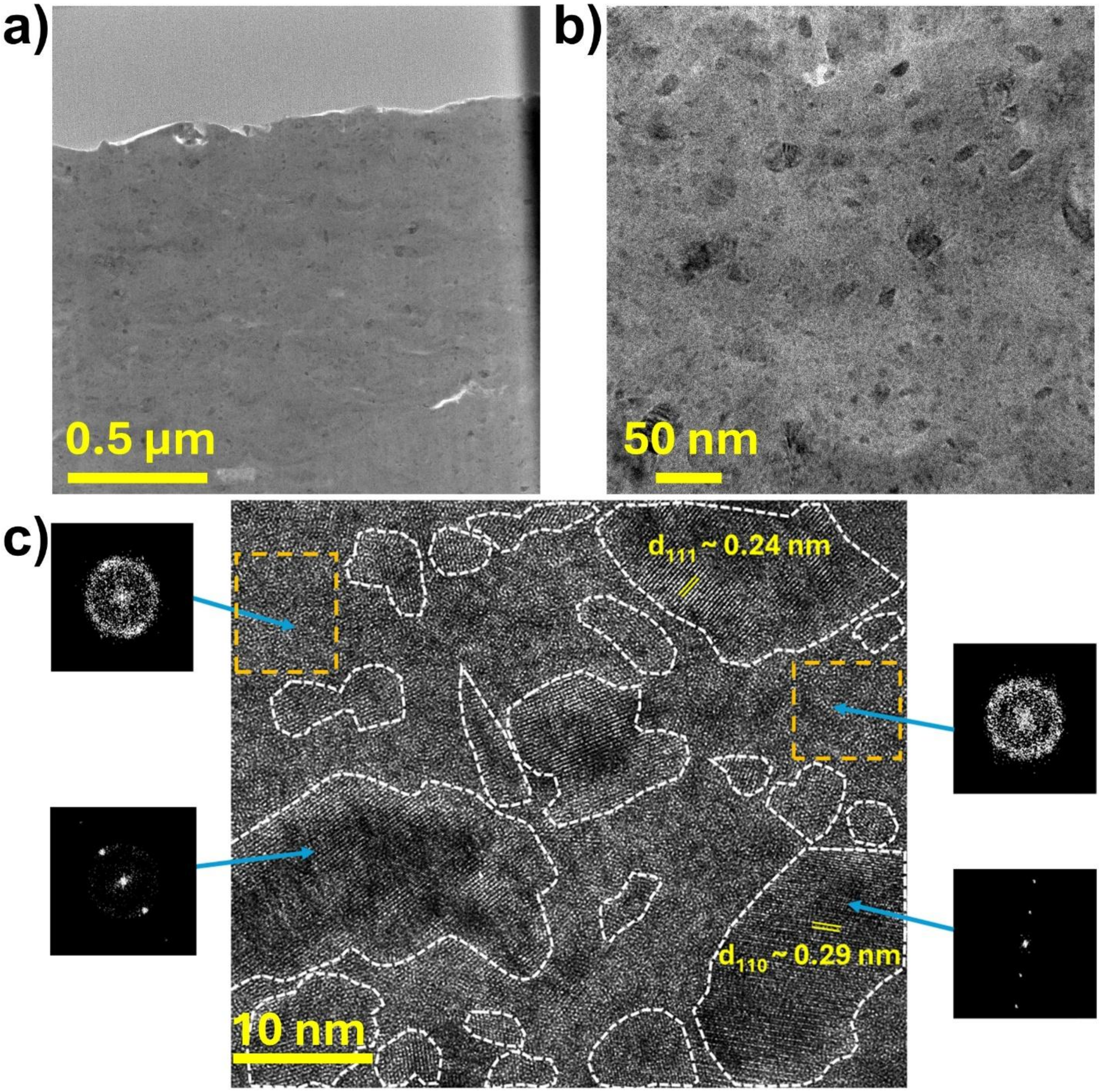

Fig.S6 HR-TEM characterization of the PZT95/5 thick film on cross sectional surface. (a,b) The cross-sectional surface view of the PZT-95/5 thick film displaying its dense microstructure at different magnifications. (c) HR-TEM images of the annealed PZT95/5 thick film cross-sectional view showing the arrangement of nanograins in a nonpolar matrix (insets display the FFT pattern for a specific area).

The obtained TEM images of the sliced film cross sectional view at different magnifications are present in Fig.S6 and they confirm the dense PZT95/5 film formation. The

magnified image present at Fig.S6b reveals the nanograins dispersion in the amorphous matrix over the whole surface area. The different interplanar spacings of the polar nanograins and the variation in the crystal planes direction of each nanograin indicates the polycrystalline nature of the prepared PZT95/5 thick film, which is aligned with the XRD results. Fig.S6c presents the Fast Fourier Transform (FFT) pattern of the highlighted surface areas from the PZT95/5 thick film. The FFT pattern on the nanograins reveals the clear formation of crystalline nature with the bright white spots on the FFT pattern, while the surrounding area of the grains exhibiting the diffused ring pattern, specifies the spreading of the polar nanograins in the non-polar matrix.

7. Elemental mapping of the AD thick film by STEM-EDS

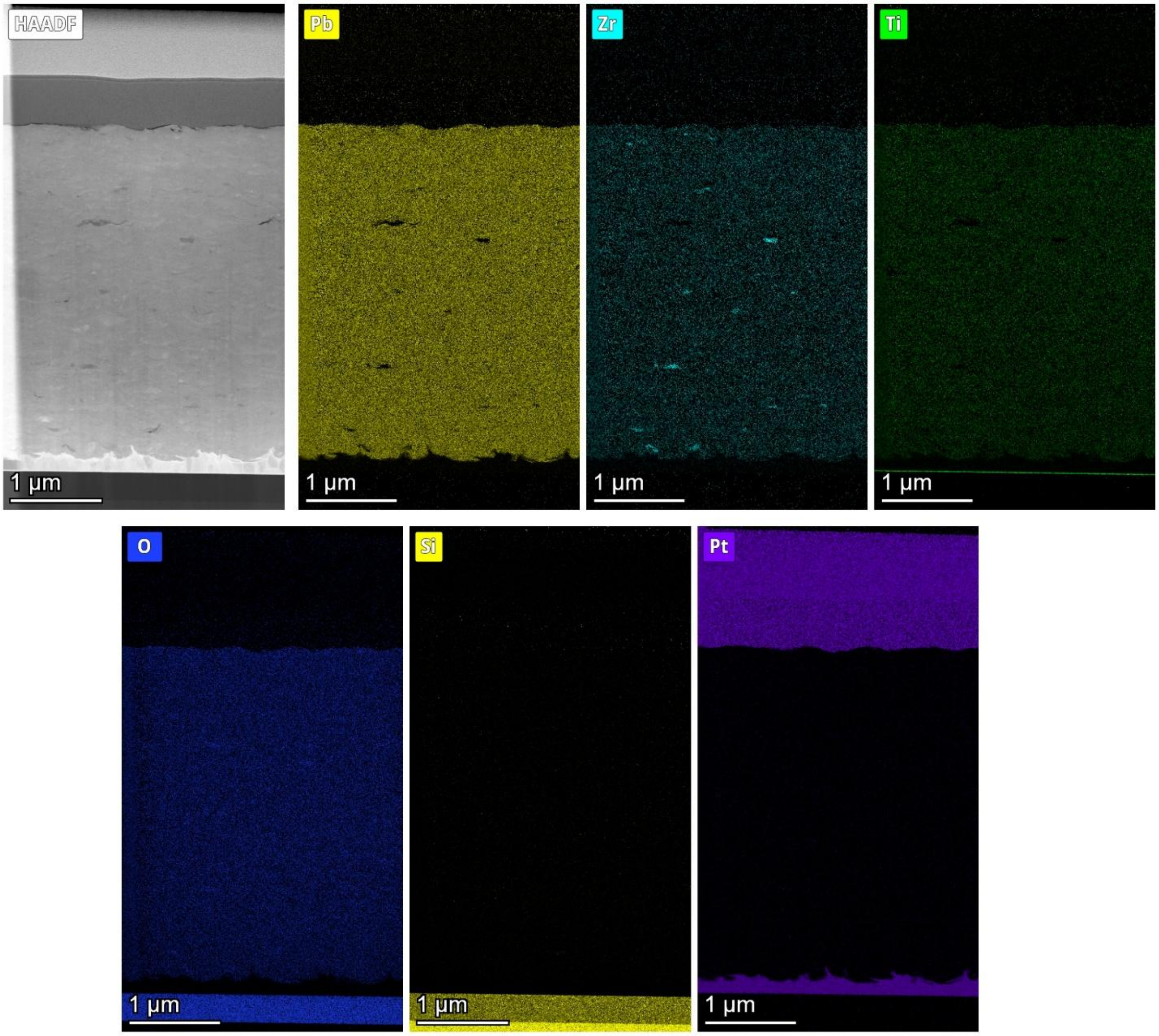

Fig.S7 High resolution cross-sectional HRTEM image and respective elemental mapping profile of the PZT95/5 thick film by EDS mapping.

Fig.S7 presents the EDS mapping from the HAADF-STEM mode, and it confirms the even distribution of all the elements such as Pb, Zr, Ti and O in the formed PZT95/5 thick film, with the clear representation for the Si and Pt elements from the substrate and deposited top electrode in this cross sectional view of the film's surface.

8. Piezo force microscopy characterization:

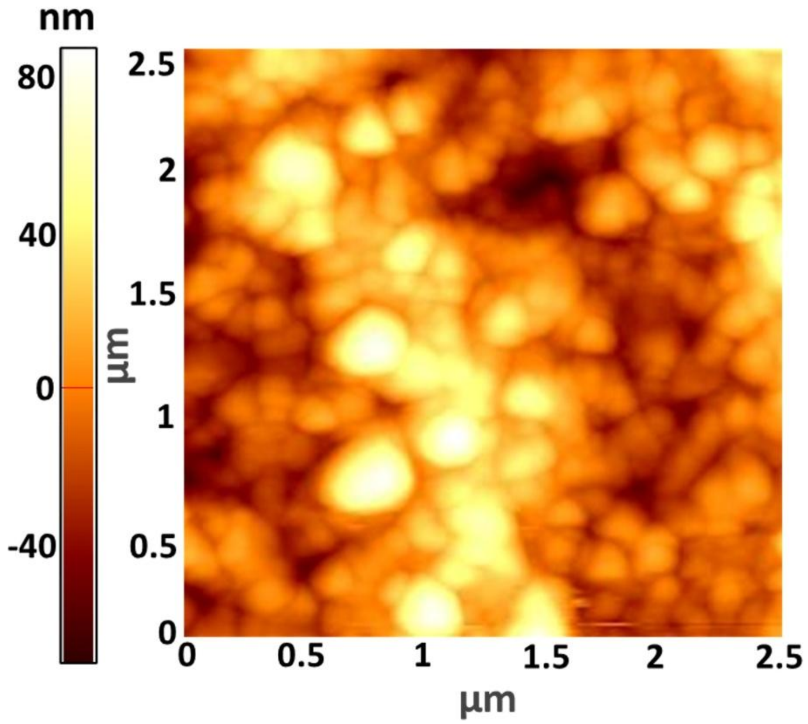

Figure S8. The PFM topography characterizations of PZT-95/5 thick films.

The Fig.S8 presents the measured topography of the sample obtained at the 2.5 × 2.5 μm^2 scanning area. The film shows slightly higher roughness (± 80 nm) due to the high kinetic energy collisions of particles with substrate in the vacuum but without any physical damage in the film surface denotes the, even film formation by the AD process.

9. Dielectric characterization:

The Ginzburg–Landau (GL) phenomenological theory proposes that materials coefficients of the free energy expansion, such as critical transition temperature drops to lower than its bulk form, when the dimension of material reduces to lower in size. The AD technique impact stress and residual stress in the thick films are biaxial compressive usually, however the stress-state through the thickness of the substrate must contain tensile stress to ensure force equilibrium. The residual stress raised biaxial strain from the AD shows impact on the dielectric properties, T_C, and ferroelectric polarization [10-13]. Also, the medium dielectric constant of thick films can lead to better E_{DBS}, due to their inverse relation between them, that is good for the improved energy storage properties.

The dispersion nature of the thick film dielectric properties were evaluated by employing the empirical expression proposed by the Uchino and Nomura [14], as given below

$$\frac{1}{\varepsilon_r} - \frac{1}{\varepsilon_m} = \frac{(T-T_m)^\gamma}{C}$$

Where ε_m is the maximum dielectric constant at phase transition temperatures, C is the constant, and γ is the diffuseness indicator. The value of γ indicates the disorder of the diffuseness in the systems, and it generally lies between $1 \le \gamma \le 2$. The normal traditional ferroelectrics have coefficient as 1, while the ideal relaxors have it as 2 [15]. The diffuseness coefficients for the present bulk and thick film samples of PZT95/05 were determined from the slopes of ln ($1/\varepsilon_r$ - $1/\varepsilon_m$) vs. ln (T - T_m) lines at 10 kHz as presented in Fig.3c and 3d. The coefficient was increased from 1.08 for bulk ceramics to 1.86 in thick film, confirming the samples enhancement in the relaxor ferroelectric nature on prepared films. The mechanically tailored polar nano regions (PNRs) formation in the film sample leads to the emergence of the RFE nature in the active samples rather than the usual heterogeneity induced relaxor characteristics. The long range ordered microdomains of the normal ferroelectric converted to dense and short range ordered nanodomains by the high kinetic energy developed during the AD technique in the film fabrication, resulting in diffusive dielectric properties of the PZT-95/5 thick films [3]. The nanograin crystallites of PZT-95/5, created by the high-speed deposition of the particles on the substrates, surrounded by the non-polar structure drastically improves the E_{DBS} and enhances the electric field dependent polarization, that is probably much clearer from the ferroelectric characterizations.

10. Ferroelectric characterization:

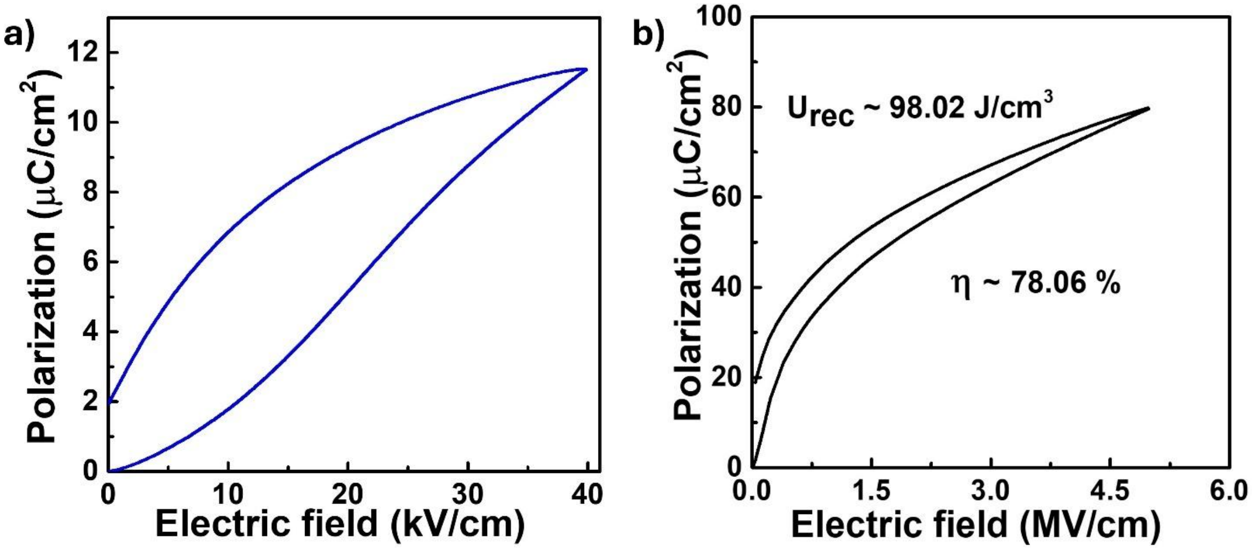

Figure S9. Ferroelectric unipolar loops of bulk PZT 95/5 ceramics and thick films. (a) Unipolar loop of bulk PZT 95/5 ceramics, upon applied 40 kV/cm electric field. (b) Unipolar loop of PZT 95/5 thick film, upon the characteristic applied electric field of 4.96 MV/cm for evaluating its energy storage properties.

The ferroelectric unipolar loop of the bulk ceramic sample was evaluated and presented in Fig.S9a. On comparing with the thick film sample properties, the bulk sample exhibits low E_{BDS} and P_{max}, with the high P_{r} reveals to be, not an ideal candidate for energy storage applications due to its traditional ferroelectric nature in bulk ceramics form, with micro grains resulting in long-range ferroelectric order. Fig.S9b presents the unipolar loop of the PZT 95/5 thick film measured under the statistically optimized electric field of 4.96 MV/cm from the Weibull distribution, to evaluate its energy storage properties. The PZT 95/5 thick film delivered recoverable energy density (U_{rec}) and efficiency (η) of 98.02 J/cm^3 and 78.06 %, respectively.

11. Charging discharging analysis:

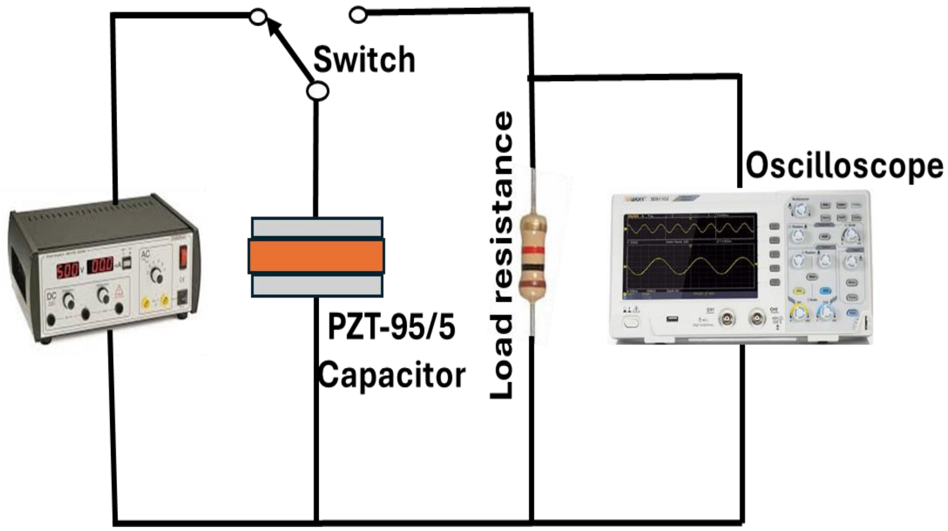

Figure S10. Schematic of the home-made setup used in the charge and discharge cycle measurements.

The schematic diagram in Fig.S10 presents the home-made setup used in the charge and discharge cycle measurements. The setup includes the high voltage power supply, metal oxide semiconductor effect transistor (MOSFET) based high speed switching circuit, an oscilloscope to observe and collect the data of charge and discharge profile.

Table S1. Recent literature on energy storage performance of ceramic thick films

Materials	Thickness (µm)	Fabrication method	U_{rec} (J/cm^3)	η (%)	E_{DBS} (MV/cm)	Ref
$Pb_{0.99}Nb_{0.02}(Zr_{0.85}Sn_{0.13}Ti_{0.02})O_3$	1.25	Sol-Gel	2.48	63.7	0.24	[16]
La doped $Pb(Zr_{0.85}Ti_{0.15})O_3$	2	Sol-gel	1.05	21	0.25	[17]
La-doped $PbZrO_3$	1	CSD	17.3	80.8	1	[18]
$BiFeO_3$- modified $(K_{0.5}Na_{0.5})(Mn_{0.005}Nb_{0.995})O_3$	1	CSD	28	90.3	2	[19]
$Pb_{0.91}La_{0.09}(Zr_{0.65}Ti_{0.35})O_3$	1	Sol-gel	28.7	57	2.17	[20]
$Pb_{0.82}La_{0.12}(Zr_{0.85}Ti_{0.15})O_3$	1	Sol-gel	38	71	2.14	[21]
0.96NBT-0.04BMT	1	PVP-solgel	40.4	54.6	2.44	[22]
<100>-oriented $Pb_{0.835}La_{0.11}(Zr_{0.85}Ti_{0.15})O_3$	1	Sol-gel	44	71	1.95	[23]
0.96(BNT)-0.04BNZ	1	Sol-gel	50.1	63.9	2.2	[24]
Fe-doped $(Na_{0.85}K_{0.15})0.5Bi_{0.5}TiO_3$	1.15	Sol-gel	33.3	51.3	2.3	[25]
Mn-doped $Na_{0.5}Bi_{0.5}TiO_3$	1.2	PVP-solgel	30.2	47.7	2.31	[26]
$Na_{0.5}Bi_{0.5}TiO_3$	1.5	Sol-gel	12.4	43	1.2	[27]
$Pb_{0.94}La_{0.04}Zr_{0.97}Ti_{0.03}O_3$	1.5	Sol-gel	18.4	54	1.4	[28]
Mn-doped $Pb_{0.91}La_{0.09}(Zr_{0.65}Ti_{0.35})O_3$	1.5	Sol-gel	30.8	68.4	1.7	[29]
$Pb_{0.85}Ba_{0.05}La_{0.10}(Zr_{0.90}Ti_{0.10})O_3$	1.5	Sol-gel	33.6	73	2.2	[30]
<100>-oriented $Pb_{0.79}Ba_{0.11}La_{0.1}(Zr_{0.9}Ti_{0.1})O_3$	1.5	Sol gel	42.3	68	2.3	[31]
(100)-oriented $Pb_{0.97}La_{0.02}(Zr_{0.95}Ti_{0.05})O_3$	1.7	Sol-gel	12.4	-	1.12	[32]
$(Pb_{0.97}La_{0.02})(Zr_{0.91}Sn_{0.04}Ti_{0.05})O_3$	1.8	Sol gel	56	45	3.71	[33]
$Pb_{0.92}La_{0.08}(Zr_{0.52}Ti_{0.48})O_3$	2	CSD	85	65	4.5	[34]
$(Pb_{0.97}La_{0.02})(Zr_{0.98}Ti_{0.02})O_3$	3.3	Sol-gel	58.1	37.3	2.8	[35]
$BaTiO_3$	1.2	Sputtering	130	76	6.5	[36]
$Pb(Zr_{0.52}Ti_{0.48})O_3$	4	AD	124	64	5.4	[3]Our work
$Pb(Zr_{0.95}Ti_{0.05})O_3$	4	AD	116	78	5.6	Present work

References:

[1] D.R. Patil, V. Annapureddy, J. Kaarthik, A. Thakre, J. Akedo, J. Ryu, Piezoelectric thick film deposition via powder/granule spray in vacuum: A review. Actuators. **9**, 59 (2020). https://doi.org/10.3390/act9030059.

[2] M. Peddigari, H. Palneedi, G.-T. Hwang, K.W. Lim, G.-Y. Kim, D.-Y. Jeong, J. Ryu, Boosting the recoverable energy density of lead-free ferroelectric ceramic thick films through artificially induced quasi-relaxor behavior. ACS Appl. Mater. Interfaces. **10**, 20720-20727 (2018). https://doi.org/10.1021/acsami.8b05347.

[3] M. Peddigari, B. Wang, R. Wang, W.H. Yoon, J. Jang, H. Lee, K. Song, G.T. Hwang, K. Wang, Y. Hou, H. Palneedi, Y. Yan, H.S. Choi, J. Wang, A. Talluri, L.-Q. Chen, S. Priya, D.-Y. Jeong, J. Ryu, Giant energy density via mechanically tailored relaxor ferroelectric behavior of PZT thick film. Adv. Mater. **35**, 2302554 (2023). https://doi.org/10.1002/adma.202302554.

[4] E. Buixaderas, C. Milesi-Brault, P. Vaněk, J. Kroupa, F. Craciun, F. Cordero, C. Galassi, Peculiar dynamics of polar states at the morphotropic phase boundary of antiferroelectric $Pb(Zr_{1-x}Ti_x)O_3$. Acta Mater. **258**, 119208 (2023). https://doi.org/10.1016/j.actamat.2023.119208.

[5] F. Cordero, E. Buixaderas, C. Galassi, Damage from coexistence of ferroelectric and antiferroelectric domains and clustering of O vacancies in PZT: An elastic and raman study. Materials. **12**, 957 (2019). https://doi.org/10.3390/ma12060957

[6] A. Ferri, A. Da Costa, J. Bauwens, Y. Pérez-Martín, A. Peláiz-Barranco, J.d.l.S. Guerra, Evidences of the ferroelectric and antiferroelectric phases coexistence in the $(Pb_{0.96}La_{0.04})(Zr_{0.95}Ti_{0.05})_{0.99}O_3$ ceramic system by probing nanoscale analyses via piezoresponse force microscopy. J. Am. Ceram. Soc. **107**, 3170-3179 (2024). https://doi.org/10.1111/jace.19611.

[7] M. Chandran, B. Tiwari, C. Kumaran, S.K. Samji, S. Bhattacharya, M.R. Rao, Integration of perovskite PZT thin films on diamond substrate without buffer layer. J. Phys. D: Appl. Phys. **45**, 202001 (2012). https://doi.org/10.1088/0022-3727/45/20/202001.

[8] R. Kumar, K. Asokan, S. Patnaik, B. Birajdar, Evolution of relaxor properties in lanthanum (La) doped barium zirconate titanate. Ferroelectrics. **517**, 8-13 (2017). https://doi.org/10.1080/00150193.2017.1369820.

[9] M. Fahad, A. Waqar, B. Kim, Effective-performance of inorganic and organic (barium zirconium titanate/polyvinylidene fluoride) piezoelectric composite for energy harvesting and self-powered smart IoT-based electronics. J. Alloys Compd. **985**, 174033 (2024). https://doi.org/10.1016/j.jallcom.2024.174033.

[10] D. Tian, P. Chen, X. Yang, B. Chu, Thickness dependence of dielectric and piezoelectric properties from the surface layer effect of $BaTiO_3$-based ceramics. Ceram. Int. **47**, 17262-17267 (2021). https://doi.org/10.1016/j.ceramint.2021.03.037.

[11] J. Silva, K. Sekhar, A. Almeida, J.A. Moreira, M. Pereira, M. Gomes, Influence of laser repetition rate on ferroelectric properties of pulsed laser deposited $BaTiO_3$ films on platinized silicon substrate. Appl. Phys. A. **113**, 379-384 (2013). https://doi.org/10.1007/s00339-013-7948-0.

[12] A. Kumar, G. Lee, Y.G. Chae, A. Thakre, H.S. Choi, G.H. Nam, J. Ryu, Induced slim ferroelectric hysteresis loops and enhanced energy-storage properties of Mn-doped $(Pb_{0.93}La_{0.07})(Zr_{0.82}Ti_{0.18})O_3$ anti-ferroelectric thick films by aerosol deposition. Ceram. Int. **47**, 31590-31596 (2021). https://doi.org/10.1016/j.ceramint.2021.08.039.

[13] N.H. Khansur, U. Eckstein, H. Ursic, M. Sadl, M. Brehl, A. Martin, K. Riess, D. de Ligny, K.G. Webber, Enhanced Electromechanical Response and Thermal Stability of 0.93 $(Na_{1/2}Bi_{1/2})TiO_3$-0.07$BaTiO_3$ Through Aerosol Deposition of Base Metal Electrodes. Advanced Materials Interfaces. **8**, 2100309 (2021). https://doi.org/10.1002/admi.202100309.

[14] K. Uchino, S. Nomura, Critical exponents of the dielectric constants in diffused-phase-transition crystals. Ferroelectrics. **44**, 55-61 (1982). https://doi.org/10.1080/00150198208260644.
[15] J. Ye, J.W. Lee, H. Song, J.H. Park, K. Kalita, M. Peddigari, J. Ryu, Highly flexible ferroelectric PZT thick films on Cu/PI foil for flexible energy storage devices. J. Energy Storage. **93**, 112321 (2024). https://doi.org/10.1016/j.est.2024.112321.
[16] Q.L. Zhao, Y.K. Wang, G.P. He, J.J. Di, L. Zhao, T.T. Su, M.Y. Zhang, Z.L. Hou, D. Wang, Energy storage and thermodynamics of PNZST thick films with coexisting antiferroelectric and ferroelectric phases. Int. J. Appl. Ceram. Technol. **18**, 154-161 (2021). https://doi.org/10.1111/ijac.13642.
[17] R. Gupta, R.P. Tandon, M. Tomar, Electrocaloric and energy storage properties of sol-gel derived lanthanum doped PZT thick films. Mater. Sci. Semicond. Process. **150**, 106970 (2022). https://doi.org/10.1016/j.mssp.2022.106970.
[18] H.J. Lee, S.S. Won, K.H. Cho, C.K. Han, N. Mostovych, A.I. Kingon, S.-H. Kim, H.Y. Lee, Flexible high energy density capacitors using La-doped $PbZrO_3$ anti-ferroelectric thin films. Appl. Phys. Lett. **112**, (2018). https://doi.org/10.1063/1.5018003.
[19] S.S. Won, M. Kawahara, L. Kuhn, V. Venugopal, J. Kwak, I.W. Kim, A.I. Kingon, S.-H. Kim, $BiFeO_3$-doped $(K_{0.5}Na_{0.5})(Mn_{0.005}Nb_{0.995})O_3$ ferroelectric thin film capacitors for high energy density storage applications. Appl. Phys. Lett. **110**, (2017). https://doi.org/10.1063/1.4980113.
[20] X. Hao, Y. Wang, J. Yang, S. An, J. Xu, High energy-storage performance in $Pb_{0.91}La_{0.09}(Ti_{0.65}Zr_{0.35})O_3$ relaxor ferroelectric thin films. J. Appl. Phys. **112**, (2012). https://doi.org/10.1063/1.4768461.
[21] Y. Zhao, X. Hao, Q. Zhang, Energy-Storage Properties and Electrocaloric Effect of $Pb_{(1-3x/2)}La_xZr_{0.85}Ti_{0.15}O_3$ Antiferroelectric Thick Films. ACS Appl. Mater. Interfaces. **6**, 11633-11639 (2014). https://doi.org/10.1021/am502415z.
[22] J. Wang, Y. Li, N. Sun, J. Du, Q. Zhang, X. Hao, $Bi(Mg_{0.5}Ti_{0.5})O_3$ addition induced high recoverable energy-storage density and excellent electrical properties in lead-free $Na_{0.5}Bi_{0.5}TiO_3$-based thick films. J. Eur. Ceram. Soc. **39**, 255-263 (2019). https://doi.org/10.1016/j.jeurceramsoc.2018.10.008.
[23] Y. Zhao, X. Hao, Q. Zhang, Enhanced energy-storage performance and electrocaloric effect in compositionally graded $Pb_{(1-3x/2)}La_xZr_{0.85}Ti_{0.15}O_3$ antiferroelectric thick films. Ceram. Int. **42**, 1679-1687 (2016). https://doi.org/10.1016/j.ceramint.2015.09.122.
[24] N. Sun, Y. Li, Q. Zhang, X. Hao, Giant energy-storage density and high efficiency achieved in $(Bi_{0.5}Na_{0.5})TiO_3$–$Bi(Ni_{0.5}Zr_{0.5})O_3$ thick films with polar nanoregions. J. Mater. Chem. C. **6**, 10693-10703 (2018). https://doi.org/10.1039/C8TC03481H.
[25] J. Wang, Y. Li, N. Sun, Q. Zhang, L. Zhang, X. Hao, X. Chou, Effects of Fe^{3+} doping on electrical properties and energy-storage performances of the $(Na_{0.85}K_{0.15})_{0.5}Bi_{0.5}TiO_3$ thick films prepared by sol-gel method. J. Alloys Compd. **727**, 596-602 (2017). https://doi.org/10.1016/j.jallcom.2017.08.169.
[26] J. Wang, N. Sun, Y. Li, Q. Zhang, X. Hao, X. Chou, Effects of Mn doping on dielectric properties and energy-storage performance of $Na_{0.5}Bi_{0.5}TiO_3$ thick films. Ceram. Int. **43**, 7804-7809 (2017). https://doi.org/10.1016/j.ceramint.2017.03.094.
[27] Y. Zhao, X. Hao, M. Li, Dielectric properties and energy-storage performance of $(Na_{0.5}Bi_{0.5})TiO_3$ thick films. J. Alloys Compd. **601**, 112-115 (2014). https://doi.org/10.1016/j.jallcom.2014.02.137.
[28] Y. Zhang, Y. Li, J. Du, N. Sun, X. Hao, H. Jiang, J. Zhai, Antiferroelectric thick film grown on metal foils with fast discharge speed and excellent energy-storage properties. J. Mater. Sci.: Mater. Electron. **30**, 11945-11951 (2019). https://doi.org/10.1007/s10854-019-01545-0.
[29] Y. Liu, X. Hao, S. An, Significant enhancement of energy-storage performance of $(Pb_{0.91}La_{0.09})(Zr_{0.65}Ti_{0.35})O_3$ relaxor ferroelectric thin films by Mn doping. J. Appl. Phys. **114**, (2013). https://doi.org/10.1063/1.4829029.
[30] H. Gao, X. Hao, Q. Zhang, S. An, L.B. Kong, Electrocaloric effect and energy-storage performance in grain-size-engineered PBLZT antiferroelectric thick films. J. Mater. Sci.: Mater. Electron. **27**, 10309-10319 (2016). https://doi.org/10.1007/s10854-016-5114-0.

[31] H. Gao, N. Sun, Y. Li, Q. Zhang, X. Hao, L.B. Kong, Q. Wang, Enhanced electrocaloric effect and energy-storage performance in PBLZT films with various Ba^{2+} content. Ceram. Int. **42**, 16439-16447 (2016). https://doi.org/10.1016/j.ceramint.2016.08.054.
[32] X. Hao, Z. Yue, J. Xu, S. An, C.-W. Nan, Energy-storage performance and electrocaloric effect in (100)-oriented $Pb_{0.97}La_{0.02}(Zr_{0.95}Ti_{0.05})O_3$ antiferroelectric thick films. J. Appl. Phys. **110**, (2011). https://doi.org/10.1063/1.3641983.
[33] X. Hao, Y. Wang, L. Zhang, L. Zhang, S. An, Composition-dependent dielectric and energy-storage properties of $(Pb,La)(Zr,Sn,Ti)O_3$ antiferroelectric thick films. Appl. Phys. Lett. **102**, (2013). https://doi.org/10.1063/1.4802794.
[34] B. Ma, Z. Hu, R.E. Koritala, T.H. Lee, S.E. Dorris, U. Balachandran, PLZT film capacitors for power electronics and energy storage applications. J. Mater. Sci.: Mater. Electron. **26**, 9279-9287 (2015). https://doi.org/10.1007/s10854-015-3025-0.
[35] Y. Wang, X. Hao, J. Yang, J. Xu, D. Zhao, Fabrication and energy-storage performance of $(Pb,La)(Zr,Ti)O_3$ antiferroelectric thick films derived from polyvinylpyrrolidone-modified chemical solution. J. Appl. Phys. **112**, (2012). https://doi.org/10.1063/1.4742128.
[36] Y. Zhao, J. Ouyang, K. Wang, M. Yuan, Y. Gao, Y. Su, H. Cheng, M. Liu, Q. Yang, W. Pan, Achieving an ultra-high capacitive energy density in ferroelectric films consisting of superfine columnar nanograins. Energy Storage Mater. **39**, 81-88 (2021). https://doi.org/10.1016/j.ensm.2021.04.010.
